# Inhibition of fusidic acid resistance through restricting conformational flexibility in domain III of EF-G

**DOI:** 10.1073/pnas.2508779122

**Published:** 2025-11-24

**Authors:** Alexandra Schindl, Megan E. Jones, Leela Ghimire, Arnout P. Kalverda, Gemma Wildsmith, Antonio N. Calabrese, Jennifer H. Tomlinson

**Affiliations:** ^a^School of Molecular and Cellular Biology, University of Leeds, Leeds LS2 9JT, United Kingdom; ^b^Astbury Centre for Structural Molecular Biology, Faculty of Biological Sciences, University of Leeds, Leeds LS2 9JT, United Kingdom; ^c^Newcastle University Biosciences Institute, Newcastle University, Newcastle upon Tyne NE2 4HH, United Kingdom

**Keywords:** FusB, Elongation Factor-G, antimicrobial resistance, NMR, conformational flexibility

## Abstract

Fusidic acid (FA) is one of few remaining antibiotics active against Methicillin-resistant *Staphylococcus aureus*. FusB confers resistance to FA by rescuing the translocation factor Elongation Factor-G (EF-G) from FA-stalled ribosome complexes. FusB induces allosteric effects on dynamics in EF-G, causing significant changes in the conformational flexibility of domain III that result in an increase in a minor, more disordered state, overcoming the steric block induced by FA. We show that restraining flexibility in the two central β-strands of EF-G domain III prevents the FusB-induced increase in this minor state population, preventing FusB-mediated release of EF-G from the ribosome and thereby reinstating FA-induced stalling of protein synthesis. We further identify a region controlling access to the minor state population, potentially pinpointing the allosteric mechanism within domain III by which FusB acts. Our findings suggest a possible region that could be targeted for rational design of an inhibitor of FusB-mediated conformational flexibility, reinstating FA sensitivity even in the presence of FusB, which could rejuvenate the efficacy of this clinically important antibiotic.

## Significance

Antibiotic resistance is a significant threat to human health, projected to cause more deaths than cancer by 2050. A lack of new drugs in the development pipeline underlines the importance of exploring alternative solutions. One potential solution is to circumvent mechanisms of resistance to current antibiotics by developing inhibitors of these mechanisms, rejuvenating the usefulness of current therapies. However, this requires a detailed understanding of existing mechanisms and the identification of druggable sites to inhibit them. This study reveals the key interactions controlling FusB-mediated resistance to fusidic acid, an important treatment for Methillicin-resistant *Staphylococcus aureus* and other *Staphylococcus* infections. By revealing these control regions, we identify a potential binding site for inhibition of the resistance mechanism.

Methicillin-resistant *Staphylococcus aureus* (MRSA) is responsible for hospital-acquired infections of the bloodstream, skin, eyes, and soft tissue (1). Fusidic acid (FA) is one of the last remaining effective antibiotic treatments for MRSA, and FA resistant MRSA poses a substantial threat to public health (2–4). A target protection mechanism is the primary source of FA resistance. The FusB family of proteins bind to the drug-target, Elongation Factor-G (EF-G), and protect EF-G from FA inhibition (5–10). Understanding the detailed molecular mechanism of resistance is crucial in developing novel therapeutics to overcome this mechanism.

As a GTPase and translocase essential in protein synthesis, EF-G undergoes large conformational changes following GTP hydrolysis, promoting the translocation of transfer RNA (tRNA) from the A to the P site on the ribosome (11, 12) before dissociating from the ribosome, allowing access for the next tRNA. Major structural rearrangements during translocation involve domains I and II moving relative to domains III to V (13), and dissociation from the ribosome requires transmission of these conformational changes from domains I and II to the other domains (14). FA binds to ribosome-bound EF-G between domains II and III and prevents EF-G release from the ribosome, blocking access for subsequent tRNAs and stalling protein biosynthesis (15). FusB binds to EF-G domains IV and V, promoting dissociation of stalled EF-G:ribosome:FA complexes but does not directly interact with domain III or FA (7). EF-G undergoes conformational changes within domains IV and V and allosterically transmitted changes in domain III dynamics upon FusB binding (16). These changes in dynamics enable FusB to confer FA resistance by increasing the population of a minor state of EF-G_C3_ (domains III to V of EF-G) with a more disordered domain III (16). Prevention of this increase in the minor state population prevents FusB from rescuing stalled ribosome:EF-G:GDP complexes, showing that these dynamic changes are important in conferring resistance (16). Elucidation of key interactions governing these FusB-induced changes in domain III dynamics will advance our understanding of target protection mechanisms of resistance and may allow the identification of a potential target site for inhibitors of the resistance mechanism to rejuvenate the efficacy of FA.

**Fig. 1.**
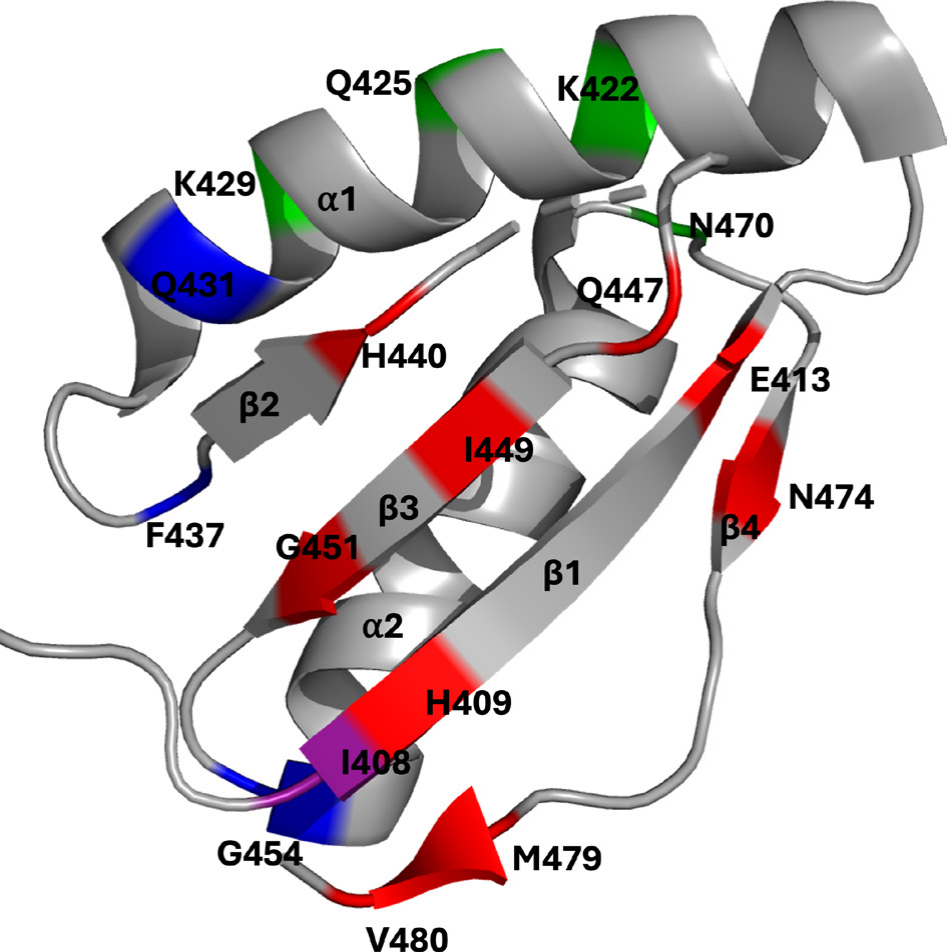
Residues substituted with cysteine to introduce disulfide bonds restricting domain III dynamics mapped onto the structure of *S. aureus* EF-G (PDB 2XEX) (17). Red: within β-sheets (I_408_C/V_480_C, H_409_C/M_479_C, E_413_C/N_474_C, H_409_C/G_451_C, E_413_C/Q_447_C, and H_440_C/I_449_C), green: within α-helices (K_422_C/N_470_C and Q_425_C/K_429_C), blue: between β-strands and α-helices (I_408_C/G_454_C and Q_431_C/F_437_C), purple: involved in more than one group.

Here, we characterize the regulation of FusB-induced dynamics in EF-G domain III. We probe the role of different regions of domain III in modulating FusB-induced changes in dynamics by engineering ten variants of EF-G and EF-G_C3_, introducing one disulfide bond per variant throughout domain III. The mutations restrain β-sheets (I_408_C/V_480_C, H_409_C/M_479_C, E_413_C/N_474_C, H_409_C/G_451_C, E_413_C/Q_447_C, and H_440_C/I_449_C), or α-helices (K_422_C/N_470_C and Q_425_C/K_429_C) or covalently link β-strands and α-helices (I_408_C/G_454_C and Q_431_C/F_437_C) (Fig. 1). Variants were selected to enable identification of interactions within domain III controlling dynamics and any hotspots that inhibit FusB-mediated dynamics changes. By comparing NMR spectra of ILVA labeled wild-type (WT) and variant EF-G_C3_, methyl CPMG relaxation dispersion experiments and fluorescence-based assays we show that trapping the second α-helix of domain III leads to a FusB-bound-like response in the absence of FusB, whereby EF-G alone is FA resistant. Conversely, restraining the two middle β-strands of domain III leads to an apo-like response in which FusB cannot confer FA resistance. We therefore identify a potential target site for the design of therapeutics to inhibit FA resistance.

## Results

To assess differences in FusB-induced changes in dynamics of EF-G_C3_ variants, NMR ILVA methyl-TROSY ^1^H-^13^C multiple-quantum Carr-Purcell-Meiboom-Gill (CPMG) relaxation dispersion experiments were performed for each variant bound to FusB and for unbound variant I_408_C/G_454_C. These experiments study residues undergoing chemical exchange on a μs-ms timescale for two states with different chemical shifts and when used with multiple field strengths can provide information on the rate of exchange, the population of each state and the difference in chemical shifts between them (18, 19). Except for variants H_409_C/G_451_C, E_413_C/Q_447_C, I_408_C/G_454_C, and Q_425_C/K_429_C data were acquired at a single field strength. Fitting data from one field strength yields unreliable parameters; hence, fitting was only performed for variants H_409_C/G_451_C, E_413_C/Q_447_C, I_408_C/G_454_C, and Q_425_C/K_429_C. However, for qualitative comparison to identify differences in dynamics effects from those in WT EF-G_C3_ a single field strength is sufficient. For each variant, ^1^H-^13^C-HMQC and ^1^H-^15^N-TROSY-HSQC spectra showed that disulfide bonds did not prevent FusB binding or cause significant structural changes (*SI Appendix*, Figs. S1–S3).

### Flexibility of the Central β-Strands Is Essential for FusB-Induced EF-G Rescue.

**Fig. 2.**
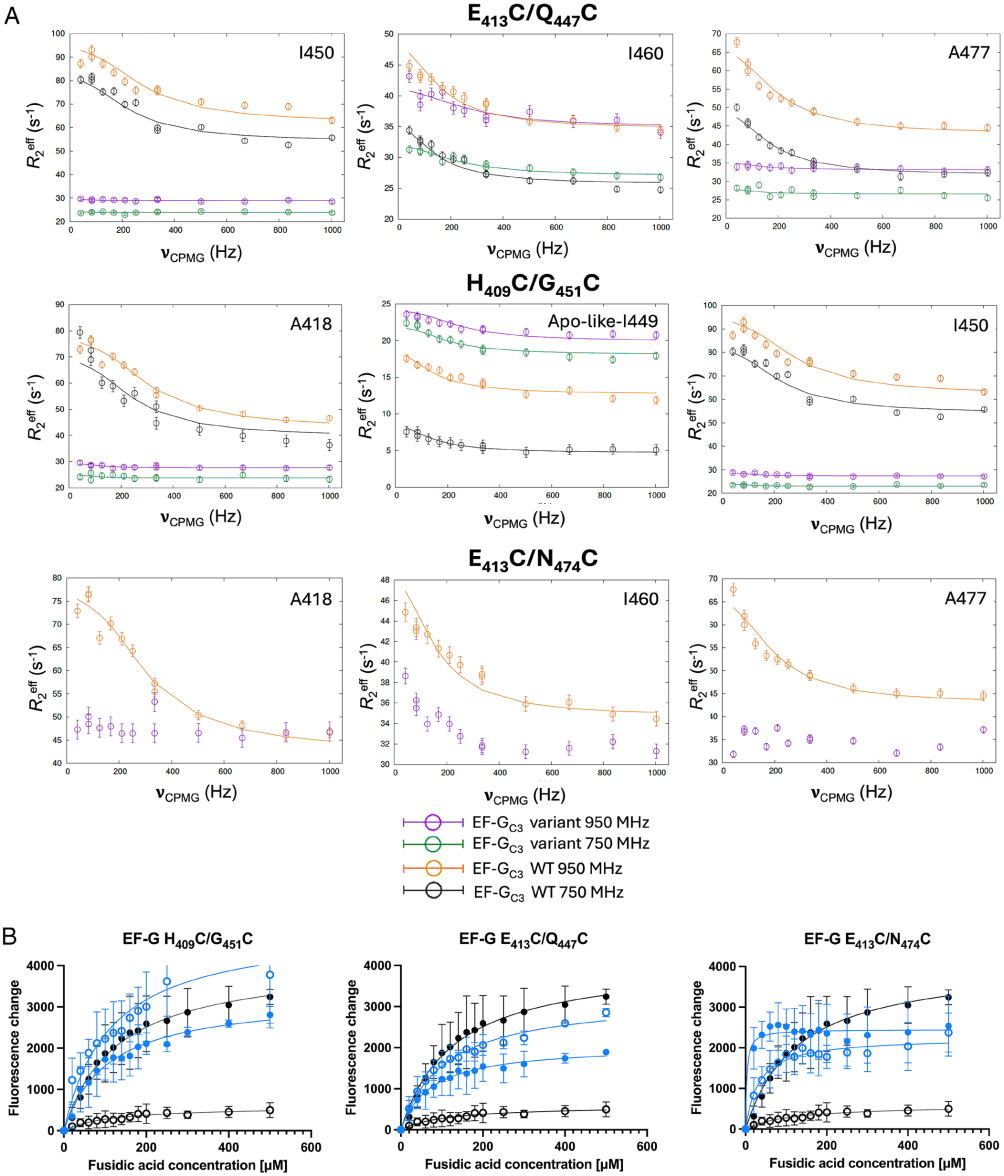
(*A*) Comparison of relaxation dispersion curves in domain III of FusB-bound EF-G_C3_ variants H_409_C/G_451_C, E_413_C/Q_447_C, and E_413_C/N_474_C at 950 MHz (purple) and 750 MHz (green) with WT EF-G_C3_:FusB at 950 MHz (orange) and 750 MHz (black), showing reduced Δ*R_2_^eff^* for the variants. Errors were determined by repeated measurements of 2 data points per experiment. *N* = 13. (*B*) Comparison of build-up of stalled ribosome:EF-G:GDP:FA complexes between WT (black) and EF-G variants (blue). Fluorescence of BODIPY-FL-GDP in response to increasing FA concentrations in the presence (open circles) and absence (closed circles) of FusB. Variants H_409_C/G_451_C, E_413_C/Q_447_C, and E_413_C/N_474_C show increased accumulation of stalled complexes in the presence of FusB compared to WT. Data points represent the mean of three replicates, error bars represent the SD. *N* = 15.

As reported previously, dispersion profiles of V_412_, A_418_, L_427_, V_448_, I_449_, I_450_, I_460_, and A_477_ in domain III show exchange on the μs-ms timescale in FusB-bound WT EF-G_C3_, while showing no or very little dispersion in apo EF-G_C3_ (16). Relaxation dispersion profiles of FusB-bound EF-G_C3_ variants restraining the central two β-strands of domain III (H_409_C/G_451_C, E_413_C/Q_447_C, and E_413_C/N_474_C) however, show greatly reduced relaxation dispersion effects in domain III compared to WT EF-G_C3_:FusB. Variants H_409_C/G_451_C and E_413_C/Q_447_C show very little dispersion overall, with E_413_C/Q_447_C preserving dynamics in I_460_ (change in *R*_2_^eff^ (Δ*R*_2_^eff^) ~7 s^−1^ compared with ~10 s^−1^ for WT) while variant E_413_C/N_474_C shows dynamics comparable to WT for I_450_ (Δ*R*_2_^eff^ ~17 s^−1^ compared with ~20 s^−1^ for WT) and I_460_ (Δ*R*_2_^eff^ ~7 s^−1^). However, all three variants show significantly reduced dispersion profiles or no dispersion for V_412_, A_418_, L_427_, I_449_, and A_477_ (Fig. 2*A* and *SI Appendix*, Fig. S4) despite large changes for WT. Relaxation dispersion data at 750 MHz in the absence of FusB also show little evidence of dispersion effects in variants H_409_C/G_451_C and E_413_C/Q_447_C, suggesting no increase in minor state population in the apo proteins (*SI Appendix*, Fig. S5).

While ^1^H-^13^C-HMQC spectra for all variants overlaid well with WT EF-G_C3_:FusB, some local chemical shift changes were apparent. For variants H_409_C/G_451_C and E_413_C/Q_447_C the I_450_ peak moves slightly toward its apo position and intensities are increased significantly (~50%) compared to FusB-bound WT (*SI Appendix*, Fig. S6) where I_450_ shows broadening. I_450_ intensities of FusB-bound H_409_C/G_451_C and E_413_C/Q_447_C are comparable to apo WT (*SI Appendix*, Fig. S6). Resonance broadening can indicate dynamics on the µs-ms timescale so this loss of broadening suggests a loss of dynamics, agreeing with the relaxation dispersion data. I_449_ shows resonances at chemical shifts matching both bound and apo WT spectra for variants H_409_C/G_451_C and E_413_C/N_474_C. Such resonance splitting also indicates a change in dynamics in the variants compared to WT EF-G_C3_:FusB. For variant E_413_C/Q_447_C, I_449_ only appears in the WT apo position. Further peaks that are broadened in the WT FusB-bound spectrum but reappear in the FusB-bound spectra of these variants are A_426_, L_430_, A_439_ (variants E_413_C/Q_447_C and H_409_C/G_451_C), and L_461_ (variant H_409_C/G_451_C). This reappearance of resonances that broaden upon FusB binding in WT EF-G_C3_ provides further evidence that these variants undergo less of a FusB-induced change in dynamics.

For variants H_409_C/G_451_C and E_413_C/Q_447_C, FusB-induced changes in dispersion profiles are reduced but not abolished completely for all residues. Therefore, relaxation dispersion data were acquired at a second field strength (750 MHz) to allow accurate parameter fitting. Previous results established that the population of a minor state of an exchange process occurring at approximately the same rate in both apo (1,100 ± 120 s^−1^) and FusB-bound (930 ± 90 s^−1^) WT EF-G_C3_ domain III, increases upon FusB binding (from 0.59 ± 0.03% to 4.7 ± 0.2%) (16). As we previously showed using hydrogen–deuterium exchange mass spectroscopy (HDX-MS), this results in greater conformational flexibility and promotes a less ordered minor conformation of domain III that drives FA resistance, potentially by preventing the inhibition of transmission of conformational changes necessary for release from the ribosome otherwise caused by FA (16). Fitting of dispersion data for FusB-bound EF-G_C3_ H_409_C/G_451_C and E_413_C/Q_447_C identified an exchange process occurring at a rate of 680 ± 130 s^−1^ with a minor state population of 0.77 ± 0.07% (variant H_409_C/G_451_C) and a rate of 1490 ± 460 s^−1^ with a minor state population of 1.08 ± 0.7% (variant E_413_C/Q_447_C). Both variants therefore show similar exchange rates to WT, albeit variant H_409_C/G_451_C has a slightly reduced and variant E_413_C/Q_447_C a slightly increased rate. Both variants also have comparable minor state populations to apo WT EF-G_C3_, showing that FusB cannot increase the minor state population in these variants as seen for WT. Taken together, the reappearance of apo resonances and the loss of FusB-induced changes in dynamics reflect an exchange process resembling that seen in WT in the absence of FusB, with FusB unable to promote an increase in the minor state population for these variants.

An exchange rate of approximately 1,000 s^−1^ should be sufficiently far from the fast exchange regime to ensure that the exchange rate and minor state population fits are not significantly affected by covariation. To confirm this, relaxation dispersion data were refitted with a range of fixed values for one parameter to determine their effect on the other (*SI Appendix*, Fig. S7). This showed that the minor state population was well defined and not significantly altered by the exchange rate for a range in which acceptable fits could be produced.

A large difference in plateau values for *R*_2_^eff^, referred to as *R*_2_^inf^, is apparent in comparisons of relaxation dispersion data between FusB-bound variant and WT EF-G_C3_ for many residues, with the species showing a higher minor state population showing a higher *R*_2_^inf^. However, the *R*_2_ for the major state is expected to be the same in each case, which would lead to similar *R*_2_^inf^ values. This difference in *R*_2_^inf^ is unlikely to be due to differences in affinity, as the greatest effects are concentrated in domain III, distant from the domains IV-V binding site (*SI Appendix*, Fig. S8). FusB-bound spectra also show no evidence of apo EF-G_C3_ peaks in the binding site, suggesting that the protein is saturated. The *R*_2_^inf^ in multiquantum relaxation dispersion data, however, has been previously shown to be influenced by the difference in ^1^H chemical shift between the major and minor states (Δω) (19–21). As previous work shows that the minor state comprises a more disordered form of domain III (16), it is likely that significant ^1^H Δωs occur in this case, causing a discrepancy in the *R*_2_^inf^ values. To assess this, ^1^H-^13^C multiquantum relaxation dispersion curves were simulated using Chemex (22), fixing the *K*_ex_ and ^13^C Δω to those obtained in WT EF-G_C3_:FusB (16) and the *R*_2_ to that observed in EF-G_C3_ E_413_C/Q_447_C:FusB, with a ^1^H Δω of either 0.02 ppm or >0.05 ppm. Simulated curves for a minor state population of 4.7%, showed increased *R*_2_^inf^ values with increased ^1^H Δω, more closely resembling the curves obtained from WT, while those with the minor state population of 0.7% resembled those obtained for the variant (*SI Appendix*, Fig. S9). This increase in *R*_2_^inf^ was enhanced by increasing the simulated ^13^C Δω or minor state population. When the ^1^H Δω was restricted to 0.02 ppm, however, no increase in *R*_2_^inf^ was observed regardless of the minor state population or ^13^C Δω. This supports our conclusion that the discrepancies between *R*_2_^inf^ values in the WT and variant relaxation dispersion data derive from effects caused by the ^1^H Δω contributing to the apparent relaxation rate, as previously reported (21). Especially at higher populations of the minor state and exchange lifetimes near a ms the induced exchange broadening as captured in *R*_2_^inf^ can be significant even with fairly modest shift differences.

**Fig. 3.**
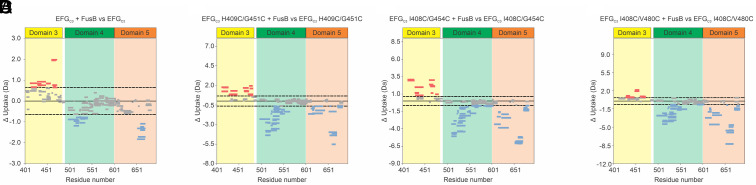
Wood’s plots showing summed differences in deuterium uptake when comparing (*A*) WT EF-G_C3_, (*B*) EF-G_C3_ H_409_C/G_451_C, (*C*) EF-G_C3_ I_408_C/G_454_C, or (*D*) EF-G_C3_ I_408_C/V_480_C, alone and in the presence of FusB. This figure was generated using Deuteros (23). Peptides colored blue or red, respectively, are protected or deprotected from exchange in the presence of FusB. Peptides with no significant difference between conditions, using a 98% CI (dotted line), are colored gray. In each case, domains IV and V (green and orange shading) show protection upon FusB binding whereas domain III (yellow shading) shows deprotection throughout the domain, suggesting the domain III minor state is similar in all three variants to that observed in WT EF-G_C3_.

To confirm that the minor state observed in the variants is similar to that observed in WT EF-G_C3_, HDX-MS was performed, comparing FusB-bound EF-G_C3_ H_409_C/G_451_C with the unbound protein. This showed a similar pattern of deprotection throughout domain III to that observed in WT upon binding FusB (Fig. 3 *A* and *B*), showing that the variant minor state is also likely composed of a more disordered form of domain III. Although the magnitude of change for EF-G_C3_ H_409_C/G_451_C appears greater than for WT, this is due to the substitutions likely reducing the minor state in the absence of FusB. Comparing the variant and WT in both apo and FusB-bound forms (*SI Appendix*, Fig. S10) shows less deprotection than the WT in each case, agreeing with the NMR data that the minor state is reduced in the variant. Our previous findings have shown that the minor state is more disordered but not fully unfolded (16), consistent with data in this study, but further characterization of the detailed conformation of the minor state is not possible from current data. Comparisons of ^13^C Δω between FusB-bound variant and WT proteins do not show a good correlation (*SI Appendix*, Fig. S11), suggesting there may be some conformational differences between the minor states, although the effects of ^1^H Δω on the fitting, discussed above, reduces the accuracy of the ^13^C Δω comparisons given different minor state populations will affect the accuracy by which ^13^C Δω can be derived. However, collectively our data show that the disulfide bond reduces the population of the minor state, but the minor state still shows the same trend as that observed in WT EF-G_C3_.

Our previous study correlated changes to domain III dynamics to impairing FusB’s ability to rescue stalled ribosome complexes (16). This was investigated further using fluorescence-based assays to follow the FA-induced build-up of stalled ribosome:EF-G:GDP complexes, using full-length EF-G harboring the same amino acid substitutions. In this assay, formation of stalled EF-G:ribosome:BODIPY-GDP:FA complexes results in increased fluorescence, while no increase is observed if stalling no longer occurs. For WT EF-G stalling occurs in response to increasing FA concentration in the absence but not the presence of FusB, as FusB rescues EF-G from FA inhibition (6). Variants H_409_C/G_451_C, E_413_C/Q_447_C, and E_413_C/N_474_C also form stalled ribosome complexes in response to increasing FA concentration in the absence of FusB, showing sensitivity to FA inhibition. However, unlike WT, all three variants show stalling in the presence of FusB (Fig. 2*B*). Therefore, these variants are not rescued from FA inhibition by FusB, despite being able to bind to FusB. For variants E_413_C/Q_447_C and H_409_C/G_451_C, the increase in fluorescence is greater in the presence than the absence of FusB, suggesting the disulfide bond potentially impairs EF-G binding to the ribosome and the modest increase in domain III minor state, while insufficient for conferring resistance, reduces this impairment of ribosome binding, resulting in increased fluorescence. To investigate this, the assay was repeated for EF-G H_409_C/G_451_C and E_413_C/Q_447_C in the presence of 5 mM DTT. No increase in fluorescence was observed in the presence of FusB, comparable to WT, showing the disulfide bond is responsible for impairing FusB-mediated FA resistance. However, in the absence of FusB, there was a greater increase in fluorescence in the presence of DTT, matching or exceeding the levels observed in WT EF-G and showing that the disulfide bond was impairing the formation of ribosome:EF-G:FA complexes, but not sufficiently to confer FA resistance (*SI Appendix*, Fig. S12).

Variants H_409_C/G_451_C, E_413_C/Q_447_C, and E_413_C/N_474_C therefore restrain the two central β-strands of EF-G domain III and appear to obstruct flexibility in this region. As a result, FusB can no longer promote an increase in the minor state of EF-G domain III and cannot rescue these variants from FA inhibition. Taken together, these data suggest that the state of the two middle β-strands determines the ability of EF-G_C3_ to increase the population of the more disordered state of domain III required for release from the ribosome in the presence of FA. Restricting the flexibility of the two middle β-strands therefore prevents changes in conformational dynamics in this region resulting in apo-like-dynamics of domain III that is capable of binding to the ribosome but is unable to be released from EF-G:ribosome:FA complexes by FusB. This region is therefore key to controlling the mechanism of FusB-induced FA resistance.

### Restraining the Second α-Helix Increases Flexibility in the Central β-Strands, Rendering EF-G Resistant to FA.

**Fig. 4.**
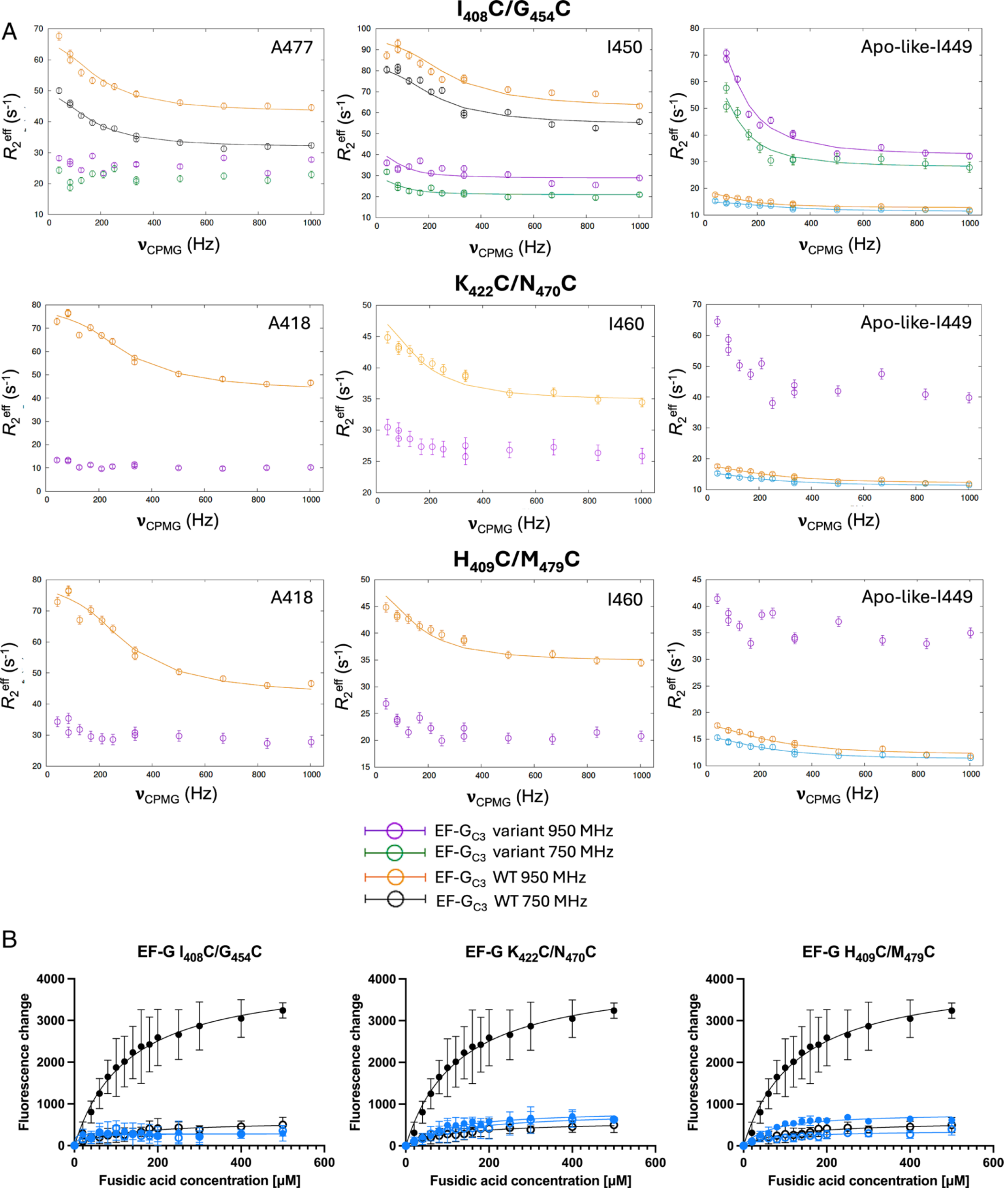
(*A*) Comparison of relaxation dispersion curves for domain III of EF-G_C3_:FusB variants I_408_C/G_454_C, K_422_C/N_470_C, and H_409_C/M_479_C at 950 MHz (purple) and 750 MHz (green) to WT EF-G_C3_:FusB at 950 MHz (orange) and 750 MHz (black) and WT apo EF-G_C3_ at 950 MHz (blue), showing greatly increased dispersion profiles for apo-like-I_449_ and reduced dispersion for other residues. Errors were determined by repeated measurements of 2 data points per experiment. *N* = 13. (*B*) Comparison of build-up of stalled ribosome:EF-G:GDP:FA complexes between WT EF-G (black) and EF-G variants (blue). Fluorescence of BODIPY-FL-GDP in response to increasing FA concentrations in the presence (open circles) and absence of FusB (closed circles). Variants, I_408_C/G_454_C, K_422_C/N_470_C, and H_409_C/M_479_C, show significantly decreased stalling in the absence of FusB compared to WT EF-G. Data points represent the mean of three replicates, error bars represent the SD. *N* = 15.

Relaxation dispersion profiles for FusB-bound EF-G_C3_ variants restraining the second α-helix of domain III (I_408_C/G_454_C, K_422_C/N_470_C, and H_409_C/M_479_C) were also compared to WT EF-G_C3_. These show consistently altered relaxation dispersion profiles, especially affecting residues I_449_, I_450_, I_460_, and A_477_ (Fig. 4*A* and *SI Appendix*, Fig. S13). I_450_, I_460_, and A_477_ are broadened in ^1^H-^13^C-HMQC spectra and produce multiple new resonances in all three variants. Relaxation dispersion profiles of I_450_ show evidence of dispersion but the increased broadening results in curves that are too noisy to be fitted. This, however, suggests an increase in dynamics for I_450_ compared with WT. A_477_ shows no dispersion in any of these variants, indicating a loss of FusB-induced dynamics for this residue. I_460_ in variants K_422_C/N_470_C and H_409_C/M_479_C shows similar dispersion to that observed in WT EF-G_C3_ (Δ*R*_2_^eff^ ~6 s^−1^ for both variants compared with ~10 s^−1^ for WT). For all three variants, I_449_ shows peak splitting, resulting in multiple resonances corresponding to both the apo and FusB-bound WT EF-G_C3_ I_449_ resonances, suggesting more than two states are accessible for this residue. The apo-like-I_449_ resonance shows significantly increased dispersion compared with both apo and FusB-bound WT spectra (Δ*R*_2_^eff^ ~48 s^−1^ for I_408_C/G_454_C, ~17 s^−1^ for K_422_C/N_470_C and ~10 s^−1^ for H_409_C/M_479_C when bound to FusB) (Fig. 4*A*). However, the FusB-bound-like I_449_ resonance shows no increase in dispersion for any of these variants compared to WT. A_418_ shows reduced dispersion profiles in all three variants, with EF-G_C3_ I_408_C/G_454_C showing a Δ*R*_2_^eff^ of ~8 s^−1^ compared with ~30 s^−1^ in WT, while K_422_C/N_470_C and H_409_C/M_479_C show even more reduced dispersion (Δ*R*_2_^eff^ of ~3 s^−1^) (Fig. 4*A*). In variant I_408_C/G_454_C A_426_ (broadened in WT EF-G_C3_:FusB) shows increased dispersion by ~15 s^−1^ compared to apo WT, but is too broad to be fitted (*SI Appendix*, Fig. S13). These variants therefore show less FusB-induced changes in dynamics in some areas of domain III but increased dispersion profiles in the central β-strands. Furthermore, K_422_C/N_470_C is the only variant where residue I_408_ is observed in the FusB-bound spectrum, suggesting loss of FusB-induced dynamics in this region. This suggests that fixing the second α-helix affects the conformational flexibility of the first β-strand, directly below the bottom of this helix, even when the location of the restraint is distant from the β-strand itself.

Relaxation dispersion experiments were repeated at 750 MHz for FusB-bound EF-G_C3_ I_408_C/G_454_C, to allow accurate parameter fitting, identifying an exchange process occurring at a rate of 570 ± 120 s^−1^ with a minor state population of 13.6 ± 3.3%. The exchange rate is slightly lower than FusB-bound WT (930 ± 90 s^−1^) but the minor state population shows a considerable increase (FusB-bound WT: 4.7 ± 0.2%). Restraining the second α-helix, therefore, increases the dynamics within the central β-sheet region, allowing a greater increase in the population of the domain III minor state.

FA inhibition assays for EF-G I_408_C/G_454_C, K_422_C/N_470_C, and H_409_C/M_479_C show no stalling in the presence of FusB, consistent with WT. However, in contrast to WT, no build-up of stalled complexes occurs in the absence of FusB (Fig. 4*B*). This indicates either that these variants can avoid stalling and hence are resistant to FA independently of FusB, or that they cannot bind to the ribosome. To investigate this, ribosome titration assays were performed for EF-G I_408_C/G_454_C and H_440_C/I_449_C in the absence of FA in which increasing fluorescence indicates ribosome:EF-G binding. These assays show an increase in fluorescence comparable with WT EF-G, confirming that both variants bind to the ribosome (*SI Appendix*, Fig. S14). Another possibility is that FA cannot bind to these EF-G variants. To test this NMR *T*_1_ρ relaxation experiments were performed for EF-G I_408_C/G_454_C in the presence of ribosomes, adding FA to EF-G I_408_C/G_454_C:ribosome complexes, as the affinity of FA for EF-G is reduced in the absence of ribosomes (6). If FA binds to EF-G I_408_C/G_454_C, a broadening and decrease in intensity for FA peaks should be observable in the presence of ribosome:EF-G I_408_C/G_454_C due to increased relaxation rates when bound to the large complex, with increasing mixing times enhancing the effect. FA peaks broadened at 10 ms mixing time with a greater loss in intensity at 200 ms mixing time in the presence of ribosome:EF-G I_408_C/G_454_C but not in experiments containing only FA, showing that FA binds to EF-G I_408_C/G_454_C:ribosome complexes (*SI Appendix*, Fig. S14). Calculating *f* scores for the for FA peaks *R*_1_ρ in the region 0.7 to 2.0 ppm shows an *f* score of 0.73 ± 0.14 in the absence of protein, reducing to 0.32 ± 0.09 in the presence of ribosome:EF-G I_408_C/G_454_C, giving a difference in *f* score of 0.41. A difference in *f* score of ≥0.2 is considered evidence of binding, showing clearly that FA binds to this EF-G variant (24).

Investigating whether the enhanced dispersion profiles for some residues are also present in the apo state, relaxation dispersion experiments were repeated for apo EF-G_C3_ I_408_C/G_454_C. ^1^H-^13^C-HMQC spectra of apo EF-G_C3_ I_408_C/G_454_C overlay well with apo WT EF-G_C3_, but show similarities to FusB-bound WT spectra for some residues (*SI Appendix*, Fig. S15). As observed in the FusB-bound spectrum, multiple I_450_ resonances appear in the apo EF-G_C3_ I_408_C/G_454_C spectrum, but none of these overlay with the chemical shift of I_450_ in apo WT EF-G_C3_ (*SI Appendix*, Fig. S16). The appearance of multiple I_450_ resonances suggests that this region undergoes increased conformational exchange in the apo state compared to WT EF-G_C3_. Relaxation dispersion profiles for apo EF-G_C3_ I_408_C/G_454_C domain III show overall an increase in dispersion compared to apo WT (*SI Appendix*, Fig. S17). I_449_ shows a Δ*R*_2_^eff^ of ~10 s^−1^ compared to ~3.5 s^−1^ in WT. V_412_, L_427_, I_450_, and I_460_ also show slightly increased dispersion compared to apo WT, indicating enhanced dynamics in this variant in the absence of FusB. Taken together, the increase in dynamics of I_449_ between the apo and FusB-bound variant I_408_C/G_454_C indicates that changes that occur upon FusB binding have been altered but not abolished. This potentially suggests an overall FusB-bound-like dynamics for apo EF-G_C3_ I_408_C/G_454_C with the caveat that for this variant, resonances in FusB-bound methyl spectra of residues A_426_, A_430_, and A_439_ show chemical shifts comparable to apo WT EF-G_C3_ spectra.

To clarify whether the population of the domain III minor state of apo EF-G_C3_ I_408_C/G_454_C is increased compared to apo WT EF-G_C3_, relaxation dispersion data were acquired at 950 and 750 MHz. Fitting of these data shows a comparable exchange rate to WT of 1440 ± 160 s^−1^ but a minor state population of 1.4 ± 0.2%. This is lower than the population for FusB-bound WT EF-G_C3_ (4.7 ± 0.2%), but higher than for apo WT EF-G_C3_ (0.59 ± 0.03%) and suggests that this increase in population is sufficient to confer FA resistance in the absence of FusB. Comparison of apo and FusB-bound dispersion curves (*SI Appendix*, Fig. S5) also shows larger dispersion effects in the FusB-bound state, consistent with FusB increasing the minor state population further.

HDX-MS data comparing EF-G_C3_ I_408_C/G_454_C in the apo and FusB-bound states (Fig. 3*C*) shows that domain III becomes deprotected in response to FusB binding, similarly to the pattern seen for WT EF-G_C3_, indicating that the minor state is therefore showing the same characteristics as those seen for WT. Comparison of apo EF-G_C3_ I_408_C/G_454_C to apo WT EF-G_C3_ also shows that domain III is deprotected in the variant compared with WT (*SI Appendix*, Fig. S18), suggesting a similar minor state is present at a higher population, as shown by the relaxation dispersion data. As the splitting of the I_449_ and I_450_^1^H-^13^C-HMQC peaks suggest more than two states can be accessed and comparison of ^13^C Δω does not produce a good correlation (*SI Appendix*, Fig. S11), these data do not allow for ruling out different conformations in the minor state or multiple minor states, but show that the same characteristics of increasing disorder throughout domain III are present, showing similar minor state traits. Therefore, the population of the minor state is key to conferring resistance to FA but a similar minor state is present in each case.

Taken together, variants I_408_C/G_454_C, H_409_C/M_479_C, and K_422_C/N_470_C restrain the flexibility of the second α-helix of domain III, resulting in a reduction in FusB-induced dynamics in the region surrounding I_408_. Through this, these variants appear to increase the flexibility of the central β-strands, as reflected in I_449_ and I_450_ on one of the central β-strands showing increased relaxation dispersion profiles and resonance broadening. This allows variants I_408_C/G_454_C, H_409_C/M_479_C, and K_422_C/N_470_C to populate the domain III minor state at an increased level in the absence of FusB, exhibiting FA resistance even in the absence of FusB.

### The Region Around G_451_ Is Central to Inhibition of FusB Rescue.

Variant H_440_C/I_449_C fixes the outer β-strand under the first α-helix to the adjacent central β-strand and is similar to the previously published variant H_438_C/G_451_C, which is closer to the region around I_408_ (6, 16). Domain III residues in FusB-bound EF-G_C3_ H_440_C/I_449_C show no significant dispersion, indicating overall decreased domain III dynamics compared with WT. However, an additional resonance is observed at the apo chemical shift of A_477_ that is not observed in FusB-bound WT spectra, I_450_ is broadened beyond detection and I_460_ is split and broadened, suggesting some conformational exchange still occurs in parts of domain III in response to FusB binding.

**Fig. 5.**
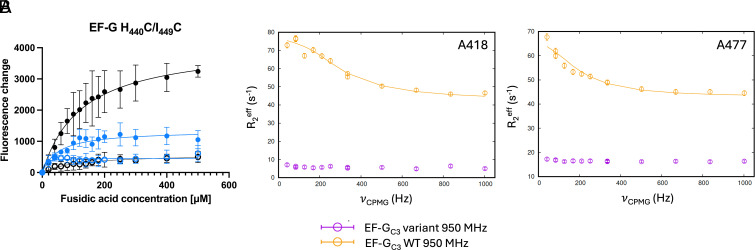
(*A*) Impact of FusB on accumulation of stalled ribosome:EF-G:GDP:FA complexes, comparing WT (black) and EF-G H_440_C/I_449_C (blue). Fluorescence of BODIPY-FL-GDP in response to increasing FA concentrations in the presence (open circles) and absence of FusB (closed circles). Variant H_440_C/I_449_C shows decreased but not abolished stalling in the absence of FusB but is rescued from FA inhibition by FusB. Data points represent the mean of three replicates, error bars represent the SD. *N* = 15. (*B*) Comparison of methyl relaxation dispersion curves in domain III of FusB-bound EF-G_C3_ H_440_C/I_449_C at 950 MHz (purple) to WT EF-G_C3_:FusB at 950 MHz (orange), showing a greatly reduced Δ*R_2_^eff^* for the variant. Errors were determined by repeated measurements of 2 data points per experiment. *N* = 13.

Both the previously published variant H_438_C/G_451_C and variant H_440_C/I_449_C show decreased stalling of ribosome:EF-G:FA complexes (Fig. 5), despite ribosome titration assays demonstrating variant H_440_C/I_449_C binding to ribosomes is comparable to WT (*SI Appendix*, Fig. S14) but unlike EF-G I_408_C/G_454_C, H_409_C/M_479_C, and K_422_C/N_470_C, stalling is not reduced to the level observed for FusB-bound WT in either case (Fig. 5). Stalling in the presence of FusB is similar to WT for variant H_440_C/I_449_C, while variant H_438_C/G_451_C shows no difference in stalling with and without FusB, indicating FusB rescues EF-G H_440_C/I_449_C but not EF-G H_438_C/G_451_C from FA inhibition (16).

This suggests that fixing domain III in the region of variant H_438_C/G_451_C yields a hybrid in dynamics such that this variant cannot be rescued from FA inhibition by FusB similar to variants H_409_C/G_451_C, E_413_C/Q_447_C, and E_413_C/N_474_C, since stalling is the same in the presence and absence of FusB. At the same time, variant H_438_C/G_451_C becomes more resistant to FA in the absence of FusB, as observed for variants I_408_C/G_454_C, K_422_C/N_470_C, and H_409_C/M_479_C, reflected by decreased stalling of ribosome complexes in the absence of FusB. By comparison, variant H_440_C/I_449_C similarly reduces but does not abolish FA-induced stalling in the absence of FusB (16), however FusB can still rescue stalled complexes. These findings suggest that fixing the outer β-strand to the adjacent inner β-strand alters flexibility of the inner β-strand such that FusB rescue is inhibited if the region around residue G_451_ is affected, as for variant H_438_C/G_451_C. The relative movement of these β-strands farther away from the hinge leading to the second α-helix, as for variant H_440_C/I_449_C, does not inhibit FusB rescue. However, both variants restrain movement of the second α-helix by fixing the middle β-strand (backbone-adjacent to the α-helix) to the outer β-strand and by this increase FA resistance, as reflected in decreased stalling in the absence of FusB and a change of dynamics of the central β-strand through broadening of I_450_ for variant H_440_C/I_449_C.

### A Small Increase in Minor State Population Is Sufficient to Confer FA Resistance.

**Fig. 6.**
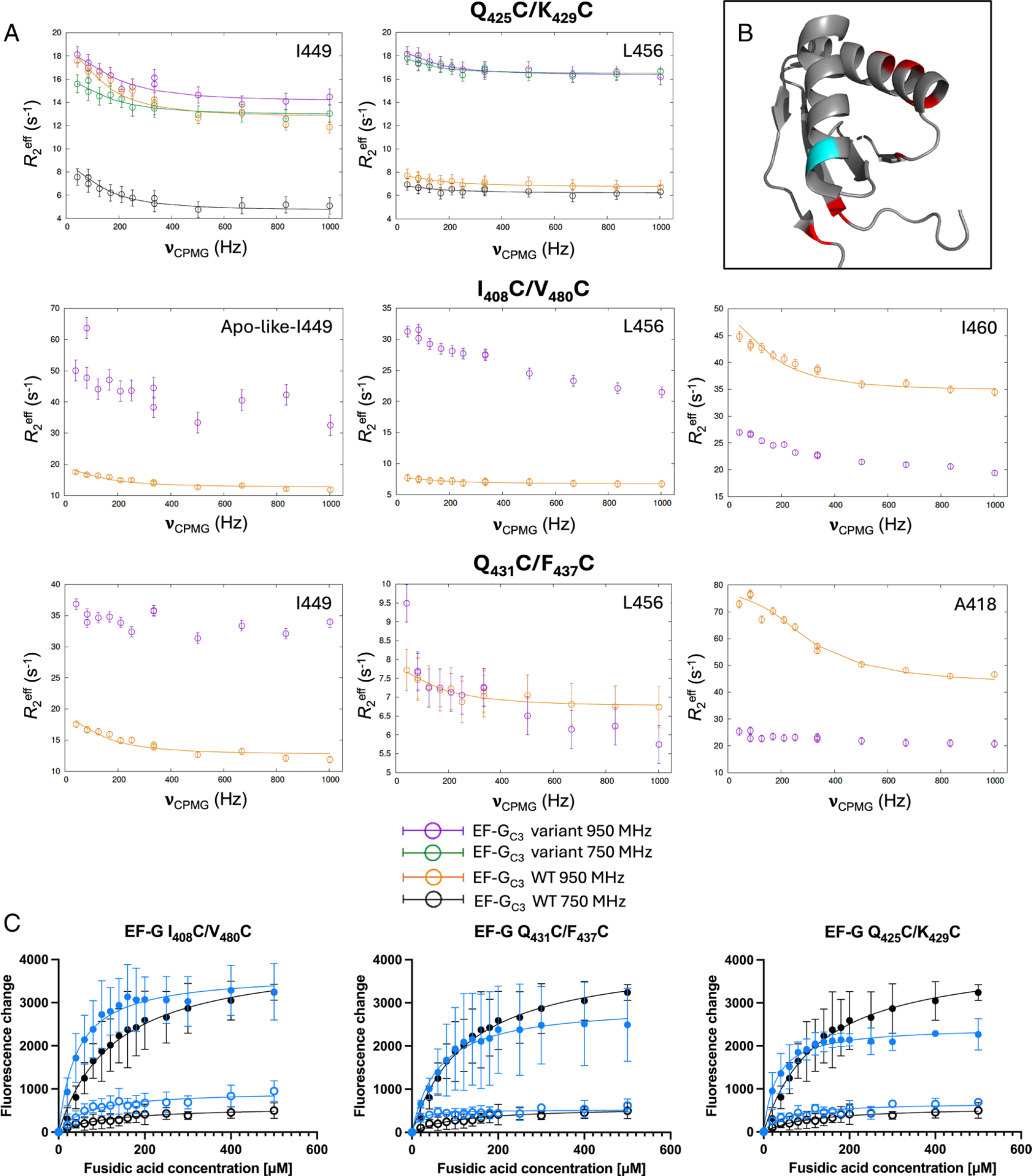
(*A*) Comparison of relaxation dispersion curves in domain III of EF-G_C3_:FusB variants K_425_C/Q_429_C, Q_431_C/F_437_C, and I_408_C/V_480_C at 950 MHz (purple) and 750 MHz (green) to WT EF-G_C3_:FusB at 950 MHz (orange) and 750 MHz (black), showing increased relaxation dispersion profiles for L_456_. Errors were determined by repeated measurements of 2 data points per experiment. *N* = 13. (*B*) The location of residues K_425_C/Q_429_C, Q_431_C/F_437_C, and I_408_C/V_480_C (red) and L_456_ (cyan) within EF-G domain III. (*C*) Comparison of the build-up of stalled ribosome:EF-G:GDP:FA complexes between WT (black) and EF-G variants (blue). Fluorescence of BODIPY-FL-GDP in response to increasing FA concentrations in the presence (open circles) and absence of FusB (closed circles). Variants K_425_C/Q_429_C, Q_431_C/F_437_C, and I_408_C/V_480_C show stalling in the absence of FusB and no stalling in the presence of FusB. Data points represent the mean of three replicates; error bars represent the SD. *N* = 15.

Relaxation dispersion data for EF-G_C3_ K_425_C/Q_429_C, Q_431_C/F_437_C, and I_408_C/V_480_C show FusB-induced dynamics for most domain III residues are decreased relative to WT, and abolished for some residues (Fig. 6*A* and *SI Appendix*, Fig. S19). I_449_ is broadened for all variants and appears in multiple new resonances for variants I_408_C/V_480_C and Q_431_C/F_437_C in ^1^H-^13^C-HMQC spectra. It shows slightly increased dispersion for variant I_408_C/V_480_C (Δ*R*_2_^eff^ ~18 s^−1^ for variant I_408_C/V_480_C, 4 s^−1^ for variant K_425_C/Q_429_C and 6 s^−1^ for variant Q_431_C/F_437_C, compared with 5.7 s^−1^ for WT). L_456_ shows increased dispersion in variants K_425_C/Q_429_C, Q_431_C/F_437_C, and I_408_C/V_480_C (Δ*R*_2_^eff^ 4.2 s^−1^, 3.8 s^−1^, and 10 s^−1^, respectively) but no dispersion in WT EF-G_C3_:FusB. Comparison of relaxation dispersion data for apo and FusB-bound Q_431_C/F_437_C (*SI Appendix*, Fig. S5) shows only small changes in dispersion profiles, consistent with only a small increase in minor state population upon FusB binding.

For EF-G K_425_C/Q_429_C, Q_431_C/F_437_C, and I_408_C/V_480_C, there is no significant difference in ribosome stalling assays compared to WT EF-G. Ribosome complexes of these variants stall upon addition of FA in the absence of FusB, while FusB confers FA resistance, despite the observed change in FusB-induced dynamics in EF-G_C3_ domain III (Fig. 6*C*).

To investigate why these variants show altered dynamics but no difference in ribosome stalling compared to WT, relaxation dispersion experiments were repeated at 750 MHz for FusB-bound EF-G_C3_ K_425_C/Q_429_C to allow accurate parameter fitting. This identified an exchange process occurring at a rate of 1,020 ± 150 s^−1^ with a minor state population of 1.33 ± 0.1%, suggesting a comparable exchange rate to WT but an approximately twofold increase in the minor state population compared to apo WT EF-G_C3_. Although the increase in population is less than for FusB-bound WT, it appears sufficient to allow release from the ribosome, as shown in ribosome stalling assays, and is comparable to the minor state population observed for apo EF-G_C3_ I_408_C/G_454_C that is sufficient for conferring FA resistance. Therefore, these data suggest a modest increase in minor state population can confer FA resistance.

HDX-MS studies of EF-G_C3_ I_408_C/V_480_C show increased deprotection in domain III upon FusB binding, suggesting the minor state is again similar to that observed in WT EF-G_C3_, but reflecting a smaller increase in population upon FusB binding to that observed in the WT (Fig. 3*D*). The appearance of multiple peaks for I_449_ in ^1^H-^13^C-HMQC spectra of this variant suggests that multiple minor state conformations might be present. However, this is limited to the region of I_449_ and the HDX-MS data show the same characteristics of increasing disorder throughout domain III, showing similar minor state traits.

## Discussion

FA resistance is conferred by mutations within EF-G or by the FusB family of proteins (3, 10, 25). FusB causes both conformational changes in EF-G_C3_ and allosterically induced changes in the dynamics of domain III (7). Previous work has pinpointed the importance of changes in EF-G_C3_ domain III dynamics for FusB-mediated FA resistance (16), with FusB binding increasing the population of a more disordered minor state that occurs natively in apo EF-G_C3_. This is sufficient to overcome FA-induced inhibition of transmission of conformational change between domains I-II and III-V required for EF-G release from the ribosome (16). Determination of specific interactions within domain III controlling these FusB-induced changes in dynamics promises a potential new target for inhibiting this resistance mechanism. Development programmes for anti-infective drugs are declining (26, 27), while antibiotic resistance is increasing (28–30). Identification of a new drug target allowing combined administration of an inhibitor of the resistance mechanism alongside FA could rejuvenate the usefulness of FA therapy at a time when FA resistance is increasing. We have determined key interactions within domain III and regions of interest that allow control of the resistance mechanism and therefore could suggest potential druggable sites to inhibit FA resistance.

**Fig. 7.**
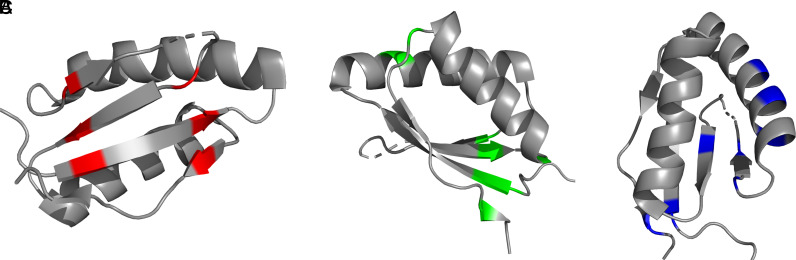
Grouping of EF-G variants according to their impact on FusB rescue of stalled complexes and domain III dynamics. (*A*) Variants H_409_C/G_451_C, E_413_C/Q_447_C, and E_413_C/N_474_C restrain the central β-strands leading to greatly reduced relaxation dispersion effects resulting from reduced minor state populations and prevent FusB from increasing the minor state population within EF-G. (*B*) Variants K_422_C/N_470_C, H_409_C/M_479_C, and I_408_C/G_454_C restrain the second α-helix and the region around residue I_408_ conferring FA resistance in the absence of FusB. Previously published variant H_438_C/G_451_C is also included in panels *A* and *B*, since overall stalling in the absence of FusB is decreased (similarly to variants of panel *B*), but FusB is unable to rescue stalled complexes (similarly to variants of panel *A*). (*C*) Variants K_425_C/Q_429_C, Q_431_C/F_437_C, and I_408_C/V_480_C show no difference to WT EF-G in FA inhibition assays despite a decrease in domain III dispersion effects. Restraint in these positions does not reduce the minor state population sufficiently to abolish FusB-mediated resistance. Variant H_440_C/I_449_C is also included in this group, since FusB can rescue stalled ribosome complexes although overall stalling is decreased.

^1^H-^13^C-HMQC spectra of variants alongside relaxation dispersion data and fluorescence assays distinguish two key regions with high importance to FusB-mediated FA resistance (summarized in *SI Appendix*, Table S1 and Fig. 7). One key region within domain III is the two central β-strands. Restraints in this region (variants H_409_C/G_451_C, E_413_C/Q_447_C, and E_413_C/N_474_C, Fig. 7*A*), prevent the FusB-induced increase in the more disordered minor state population important for allowing EF-G release from the ribosome in the presence of FA. Instead, the minor state population remains close to that of apo WT EF-G_C3_ when bound to FusB. For these variants therefore FusB-mediated resistance becomes ineffective, preventing FusB-mediated release from the ribosome and reinstating FA-induced stalling of protein synthesis. This region therefore appears to be important in controlling the mechanism of resistance and hence could be a potential target site for inhibition of FA resistance. The other key region lies at the bottom of the second α-helix and the beginning of the first β-strand which appears also to control flexibility of the central β-strands. Restraints of this region (variants K_422_C/N_470_C, H_409_C/M_479_C, and I_408_C/G_454_C, Fig. 7*B*), lead to a FusB-bound-like state that is resistant to FA regardless of the presence of FusB. This is characterized by an increase in the minor state(s) population in the absence of FusB, conferring resistance to FA, and FusB binding increases this minor state(s) population further in variant I_408_C/G_454_C. As well as identifying a key region controlling domain III dynamics, these variants reinforce the importance of the central β-strands in controlling FA resistance, as increasing dynamics in this area confers resistance in the absence of FusB, and potentially pinpoint the mechanism by which FusB acts.

The crystal structure of *T. thermophilus* EF-G in FA stalled ribosome complexes shows FA in close proximity above the second α-helix of domain III, slightly between both helices (31). Based on the findings that dynamics within the middle β-strands are required for release of stalled EF-G from the ribosome and fixing the second α-helix in place such that the flexibility of the β-strands is conserved prevents FA-induced stalling, it seems plausible that FA inhibits dynamics within the second α-helix which would otherwise allow dynamics within the region of I_408_ and promote the flexibility of the two middle β-strands, allowing conformational flexibility within this region. The hybrid behavior of variant H_438_C/G_451_C from our previous study supports this hypothesis, showing increased FA resistance alongside resistance to FusB rescue. This is contrasted with variant H_440_C/I_449_C, fixing the same β-strands as H_438_C/G_451_C but further from the region around I_408_, showing increased FA resistance, while preserving FusB-mediated rescue of stalled complexes. This indicates a switch in dynamics centered in the region of I_408_ and G_451_, which are adjacent to each other on the central β-strands.

Restricting conformational dynamics of the region around I_450_ appears paramount in preventing FusB-induced FA resistance, since EF-G_C3_ H_409_C/G_451_C and E_413_C/Q_447_C were the only variants showing no broadening of I_450_ in ^1^H-^13^C-HMQC spectra, but conversely showed increased intensity compared to WT EF-G_C3_:FusB. This correlates with loss of FusB-induced FA resistance. This is exceptional, since for all other variants I_450_ is broadened or split, indicating decreased dynamics in this region for variants H_409_C/G_451_C and E_413_C/Q_447_C and increased dynamics for all others. I_450_ sits central in the middle of domain III and is not solvent exposed, thereby perhaps being central to dynamics within domain III. Given that two glycine residues, that are not located in a structural bend, sit consecutive to I_450_, it is plausible that these residues allow increased flexibility within this region. This central β-strand region, therefore, could act as a potential binding site for a drug to inhibit FusB-mediated FA resistance.

This study has identified the region within EF-G domain III important in regulating FusB-induced dynamics and is consistent with previously published results by matching the population of a minor state of EF-G to overcoming FA-induced stalling (16). Allosteric effects induced by FusB have been disrupted or altered by the presence of restraints in EF-G domain III and we have identified key sites important in controlling FA resistance. The differing effects of restraining different regions of domain III have highlighted the key dynamics within domain III for conferring FA resistance and have shown that a small increase in minor state population is sufficient to allow FA resistance. We have therefore identified a possible druggable site to inhibit this important antibiotic resistance mechanism while also identifying the molecular switch governing FusB-induced dynamics of EF-G domain III.

## Materials and Methods

### Protein Overexpression and Purification.

All proteins were expressed and purified as previously described (7) without specific labeling. EF-G_C3_ was also IVLA methyl ^13^C-^1^H labeled on a uniformly ^12^C-^2^H-^15^N background. Additional details are available in *SI Appendix*, *SI Materials and Methods*.

### NMR.

All protein-detected NMR spectra were acquired using 250 µM EF-G_C3_^1^H-^13^C labeled Ala-Cβ, Ile-Cδ, Leu-Cδ, and Val-Cγ in a ^2^H-^12^C background, saturated with 350 µM nonisotopically enriched FusB where appropriate, in 20 mM Tris-HCl, 300 mM NaCl, pH 8.0. Spectra were recorded on Bruker Avance spectrometers at 950 or 750 MHz, equipped with cryoprobes (TXO 5-mm or TCl 3-mm at 950 MHz, TCl 5-mm at 750 MHz). Spectra were processed using nmrPipe (32) and visualized using CCPN Analysis (33) on the NMRbox platform (34).

### NMR CPMG Relaxation Dispersion.

NMR methyl relaxation dispersion experiments were acquired at 950 and 750 MHz at 30 °C using the pulse program of Korzhnev et al. (19) with a 24 ms *T*_2_ mixing time and CPMG field strengths of 41.8, 83.8 (repeated), 126.1, 168.6, 210.4, 251.9, 335.4 (repeated), 502.0, 668.7, 835.4, and 1002.0 Hz. Errors were estimated from repeated CPMG field strengths. Resonances with a Δ*R*_2_^eff^ ≥1 s^−1^ were identified as showing dispersion. Overlapped peaks or those for which the systematic variation in *R*_2_^eff^ was greater than the overall Δ*R*_2_^eff^ were discounted. Resonances for which peak height was reduced to noise level for some data points were noted as showing dispersion, but excluded from fitting since no accurate *R*_2_^eff^ values could be obtained. Data were fitted to a standard two-state exchange model using CATIA (35). A global fit to a single *K*_ex_ and pB was performed for residues within domain III listed in *SI Appendix*, Table S2.

### *T*_1_ρ NMR Experiments.

All *T*_1_ρ experiments were performed at 25 °C using a 600 MHz Bruker Avance spectrometer equipped with a cryoprobe. Samples were composed of 50 μM FA in the presence or absence of 1 μM 70S *Escherichia coli* ribosomes (NEB) and 1 μM EF-G I_408_C/G_454_C in ribosome binding buffer (50 mM Tris-HCl, 70 mM NH_4_Cl, 30 mM KCl, 7 mM MgCl_2_, 10% D_2_O, pH 7.5).

### FA Inhibition Assays.

Fluorescence-based assays measuring the build-up of stalled ribosome complexes (6) were performed in triplicate. Samples were produced in ribosome binding buffer containing 1 µM *E. coli* ribosomes (NEB), 0.1 µM BODIPY-FL-GDP (Invitrogen), and either 1 µM WT EF-G, 1 µM EF-G H_440_C/I_449_C, 1 µM EF-G Q_431_C/F_437_C, 1 µM K_425_C/Q_429_C, 1 µM E_413_C/N_474_C, 2 µM I_408_C/V_480_C, 3 µM E_413_C/Q_447_C, 10 µM I_408_C/G_454_C, 10 µM H_409_C/G_451_C, 10 µM K_422_C/N_470_C or 10 µM H_409_C/M_479_C in the presence and absence of 5x molar excess of FusB with respect to EF-G. FA was titrated over the range 0 to 500 μM. Fluorescence was measured with excitation and emission wavelengths of 485 nm and 520 nm, respectively, at 37 °C using a Hidex sense microplate reader. Assays were repeated in the presence of 5 mM DTT for EF-G WT, H_409_C/G_451_C, and E_413_C/Q_447_C.

### Ribosome Binding Assay.

Ribosome binding assays were conducted as described for the FA inhibition assay except that FA was omitted and ribosomes were titrated over the range of 0 to 2.3 μM.

### HDX-MS.

HDX-MS experiments were carried out using an automated HDX robot (LEAP Technologies, Fort Lauderdale, FL) coupled to an M-Class Acquity LC and HDX manager and Synapt G2-Si (Waters Ltd., Wilmslow, Manchester, UK). Additional details are available in *SI Appendix*, *SI Materials and Methods*.

## Supplementary Material

Appendix 01 (PDF)

## Data Availability

FA inhibition assay, ribosome titration assay data and relaxation dispersion data are available in the Open Science Forum (https://osf.io/ey8ad) (36), (https://osf.io/hrvjf) (37) and (https://osf.io/c6yz7/) (38) respectively. Mass spectrometry proteomics data have been deposited to the ProteomeXchange Consortium via the PRIDE (39) partner repository (PXD062993).
