# Supplementary material for: Inhibition of fusidic acid resistance through restricting conformational flexibility in domain III of EF-G
Source: Proc Natl Acad Sci U S A. 2025 Nov 24;122(48):e2508779122. doi: 10.1073/pnas.2508779122 (PMC12685103; doi:10.1073/pnas.2508779122)
Supplement: Supplementary file 1 — Appendix 01 (PDF) [file pnas.2508779122.sapp.pdf]

Supplementary Information

Inhibition of fusidic acid resistance through restricting conformational flexibility in domain III of EF-G

Alexandra Schindl,[a,b] Megan E. Jones[a,b] Leela Ghimire,[c] Arnout P. Kalverda,[a,b] Gemma Wildsmith,[a,b] Antonio N. Calabrese[a,b], Jennifer H. Tomlinson *[a,b]

[a]*School of Molecular and Cellular Biology, University of Leeds, Leeds LS2 9JT, United Kingdom*
[b]*Astbury Centre for Structural Molecular Biology, Faculty of Biological Sciences, University of Leeds, Leeds LS2 9JT, United Kingdom*
[c]*Newcastle University Biosciences Institute, Newcastle University, Newcastle upon Tyne NE2 4HH, United Kingdom*
**Corresponding author* ✉ *J.H.Tomlinson@leeds.ac.uk*

Supplementary Materials and Methods

Site directed mutagenesis

All mutations were introduced using the Quikchange Multi site directed mutagenesis kit (Agilent) according to the instructions of the manufacturer. The primers used are shown in **Table S3**. Successful introduction of mutations was confirmed by Sanger sequencing (Genewiz) using T7 and T7terminal universal sequencing primers as well as the primers listed in **Table S3** for sequencing of *fusA*.

Protein overexpression and purification

All proteins were expressed and purified as described previously.[1,2] *E.coli* BL21 Gold cells harbouring either pET-3a-*fusA* or pET-28a-*fusB* plasmid were cultured in LB broth for unlabelled proteins at 37 °C, 200 rpm until an OD_{600nm} of ~0.6 was reached. IPTG was added to a final concentration of 1 mM and cells were cultured at 25 °C for FusB or 18 °C for EF-G at 200 rpm overnight. For IVLA methyl detected experiments, *E. coli* BL21 Gold cells harbouring pET-29b-fusAC3 plasmid were cultured in 100 % ^{2}H M9 minimal media containing 3 g/l ^{12}C-^{2}H glucose and 1 g/l ^{15}N ammonium chloride at 37 °C, 200 rpm until an OD_{600nm} of ~0.7 was reached. Then 120 mg/l 3-methyl-^{13}C-^{1}H, ^{2}H α-ketoisovaleric acid sodium salt, 60 mg/l methyl-^{13}C-^{1}H, ^{2}H α-ketobutyric acid sodium salt, 2.5 g ^{2}H succinic acid and 0.8 g/l methyl-^{13}C-^{1}H, ^{2}H L-alanine was added and the

culture was incubated at 37 °C with shaking at 200 rpm for 60 min to exhaust non-methyl labelled precursors. Protein expression was then induced by adding 1 mM IPTG and cultures were incubated at 18 °C, 200 rpm overnight. For all expression cultures, cells were harvested by centrifugation at 4500 x*g*, 4°C for 30 min.

Cells were lysed by sonication on ice following resuspension in lysis buffer (50 mM NaH_2PO_4, 10 mM imidazole, 300 mM NaCl, pH 8.0) and addition of benzonase (Merck) and Complete EDTA-free protease inhibitors (Roche). The lysate was cleared by centrifugation at 11000 x*g*, 4 °C for 20 min. Proteins were purified using Ni-NTA affinity columns at 4°C, washing the column with 8 x column volume lysis buffer, then 8 x column volume lysis buffer containing 20 mM imidazole before eluting in lysis buffer containing 250 mM imidazole. Thereafter, proteins were dialysed overnight in 1 l 20 mM TrisHCl, 300 mM NaCl, pH 8.0 (with additional 1 mM DTT for EF-G) and further purified using size exclusion columns (S75 or S200) in 20 mM TrisHCl, 300 mM NaCl, pH 8.0 at 4 °C as previously described.[1-3]

Hydrogen-deuterium exchange mass spectrometry (HDX-MS). HDX-MS experiments were carried out using an automated HDX robot (LEAP Technologies, Fort Lauderdale, FL, USA) coupled to an M-Class Acquity LC and HDX manager (Waters Ltd., Wilmslow, Manchester, UK). Samples were prepared with 8 µM of EF-G_{C3}, $H_{409}C/G_{451}C$ variant, $I_{408}C/G_{454}C$ variant, or $I_{408}C/V_{480}C$ variant in the apo state, and with the addition of 10 µM FusB. All proteins were diluted in equilibrium buffer (10 mM potassium phosphate, 300 mM sodium chloride, pH 8). For each condition, 5 µl of sample was mixed with 95 µl of deuterated buffer (10 mM potassium phosphate, 300 mM sodium chloride, pD 8.0, 82% D_2O) and incubated for 0, 0.5, 5, or 30 minutes at 4°C, with each time point being performed in triplicate. Once the labelling reaction was complete, samples were quenched by mixing 75 µl of labelled solution with 75 µl of quench buffer (10 mM potassium phosphate, 300 mM sodium chloride, 200 mM TCEP, pH 2.2). 95 µl of the quenched reaction was injected onto an immobilised pepsin column (Enzymate pepsin column 300 Å, 5 µM, 2.1 mm x 30 mm, Waters Ltd., Wilmslow, Manchester, UK) at 20°C. A VanGuard Pre-column Acquity UPLC BEH C18 (130 Å ,1.7 µm, 2.1 mm × 5 mm, Waters Ltd., Wilmslow, Manchester, UK) was used to entrap peptides for 3 minutes in 0.3% formic acid in water. Peptides were then passed through a C18 column (Acuity UPLC BEH C18 column, 130 Å, 1.7 µm × 1

mm x 100 mm, Waters Ltd., Wilmslow, Manchester, UK) and separated using a 12 minute 5-40% (v/v) acetonitrile (0.1% v/v formic acid) in H_2O (0.3% v/v formic acid) at 40 μl min^{-1}. Resultant peptides were infused into a Synapt G2Si mass spectrometer (Waters Ltd., Wilmslow, Manchester, UK) using the $HDMS^E$ operating mode. Ion mobility was used to separate peptides before fragmentation by collision induced dissociation (CID). The HDX data were analysed using PLGS (v3.0.2) and DynamX (v3.0.0) software. Peptides were filtered in DynamX using specified parameters: minimum intensity: 10,000, minimum products per amino acid: 0.3, maximum sequence length: 25, maximum parts-per-million (ppm) error: 10, file threshold: 3. Deuteros 2.0 [5] was used to visualise the data and identify statistically significant increases/decreases in deuterium uptake between states.

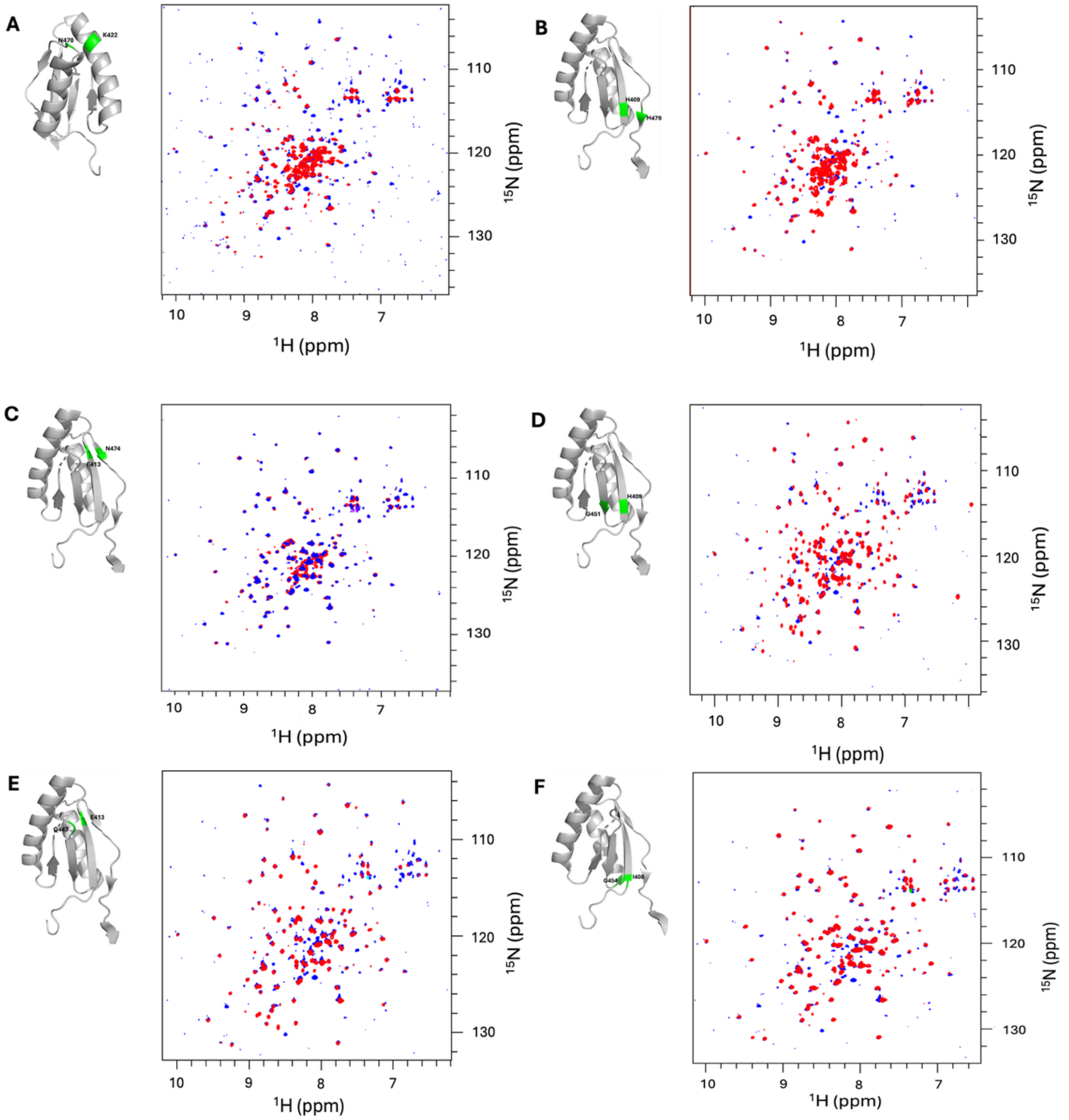

Figure S1. Mapped mutations inserted into EF-G to disrupt FusB induced dynamics without preventing binding or conformational changes. Locations of mutations are shown in green on the structure of domain III. ^{1}H-^{15}N-TROSY-HSQC spectra for each variant bound to FusB (red) are overlaid with spectra of WT EF-G$_{C3}$ bound to FusB (blue), showing few chemical shift changes. Peaks absent from spectra are alanine residues that were not ^{15}N labelled in these samples due to the use of methyl-^{13}C-^{1}H, ^{2}H L-alanine for labelling CH$_{3}$-groups, which did not include ^{15}N-labelling. Hence, all alanines are missing from spectra of variants. Spectra indicate that conformational changes and FusB binding are not perturbed by the mutations. **(A)** K$_{422}$C/N$_{470}$C, designed to fix the top of the second α-helix. **(B)** H$_{409}$C/M$_{479}$C, designed to fix the beginning of the first β-strand to the fifth β-strand-like structural element. For both K$_{422}$C/N$_{470}$C and H$_{409}$C/M$_{479}$C all FusB-bound peaks are visible with no evidence of

apo EF-G_{C3} peaks. There is a slight suggestion of additional peaks indicating some additional disorder but this is possibly due to an increased population of the domain III minor state in these variants. **(C)** $E_{413}C/N_{474}C$, designed to fix the end of the first β-strand to the fourth β-strand-like structural element. **(D)** $H_{409}C/G_{451}C$, designed to fix the central β-strands at the beginning of the first β-strand and the end of the third β-strand. **(E)** $E_{413}C/Q_{447}C$, designed to fix the end of the first β-strand to the beginning of the third β-strand. In addition to all FusB-bound peaks, some apo peaks from residues in domain III are visible in the spectrum of this variant, which is likely due to the reduced FusB-induced dynamics in this variant meaning that peaks that broaden in the WT protein are still visible. No apo peaks are visible in domains IV or V so this is unlikely to reflect incomplete saturation with FusB. **(F)** $I_{408}C/G_{454}C$, designed to fix the second α-helix to the first β-strand below.

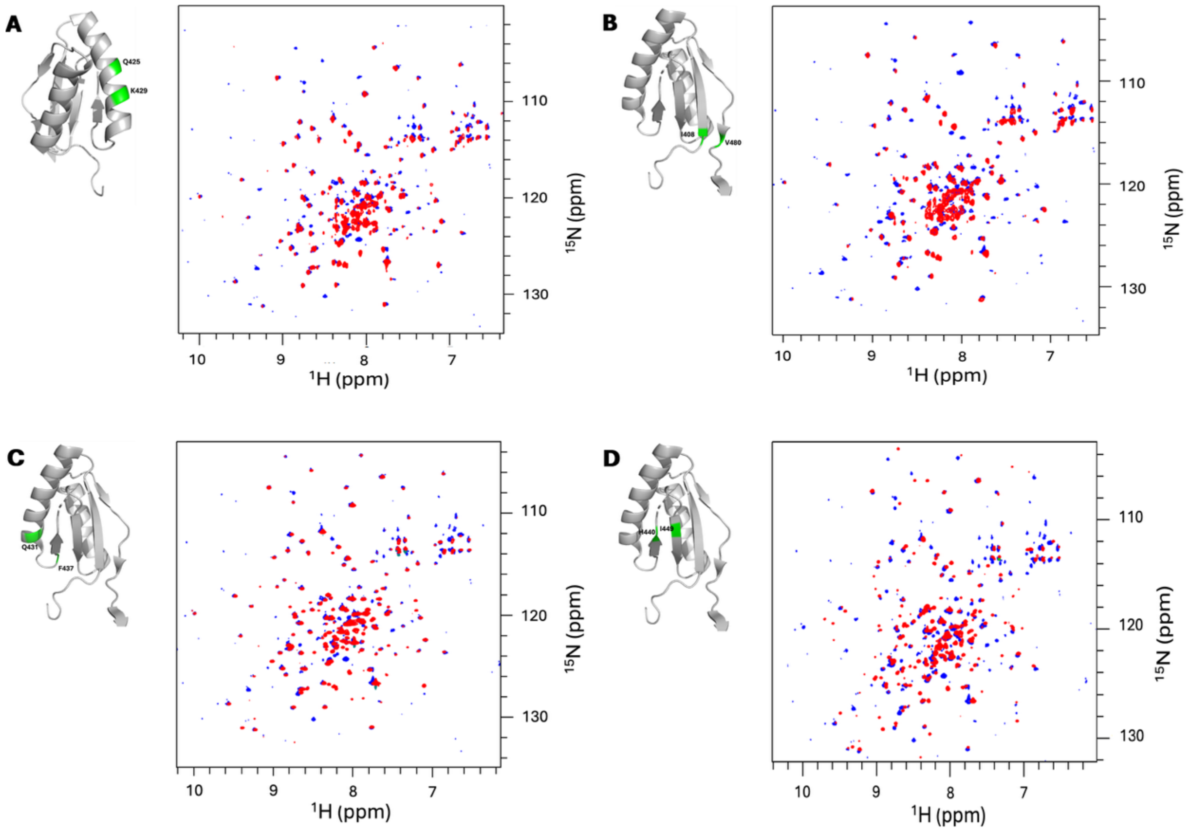

Figure S2. Mapped mutations inserted into EF-G to disrupt FusB induced dynamics without preventing binding or conformational changes. Locations of mutations are shown in green on the structure of domain III. ^{1}H-^{15}N-TROSY-HSQC spectra for each variant bound to FusB (red) are overlaid with spectra of WT EF-G_{C3} bound to FusB (blue), showing few chemical shift changes. Peaks absent from spectra are alanine residues that were not ^{15}N labelled in these samples due to the use of methyl-^{13}C-^{1}H, ^{2}H L-alanine for labelling CH_3-groups, which did not include ^{15}N-labelling. Hence, all alanines are missing from spectra of variants. Spectra indicate that conformational changes and FusB binding are not perturbed by the mutations. **(A)** $K_{425}C/Q_{429}C$, designed to intra-helically fix the first α-helix. **(B)** $I_{408}C/V_{480}C$, designed to fix the very beginning to the very end of domain III. **(C)** $Q_{431}C/F_{437}C$, designed to fix the first α-helix to the second β-strand below. **(D)** $H_{440}C/I_{449}C$, designed to fix the middle of the second β-strand to the third β-strand.

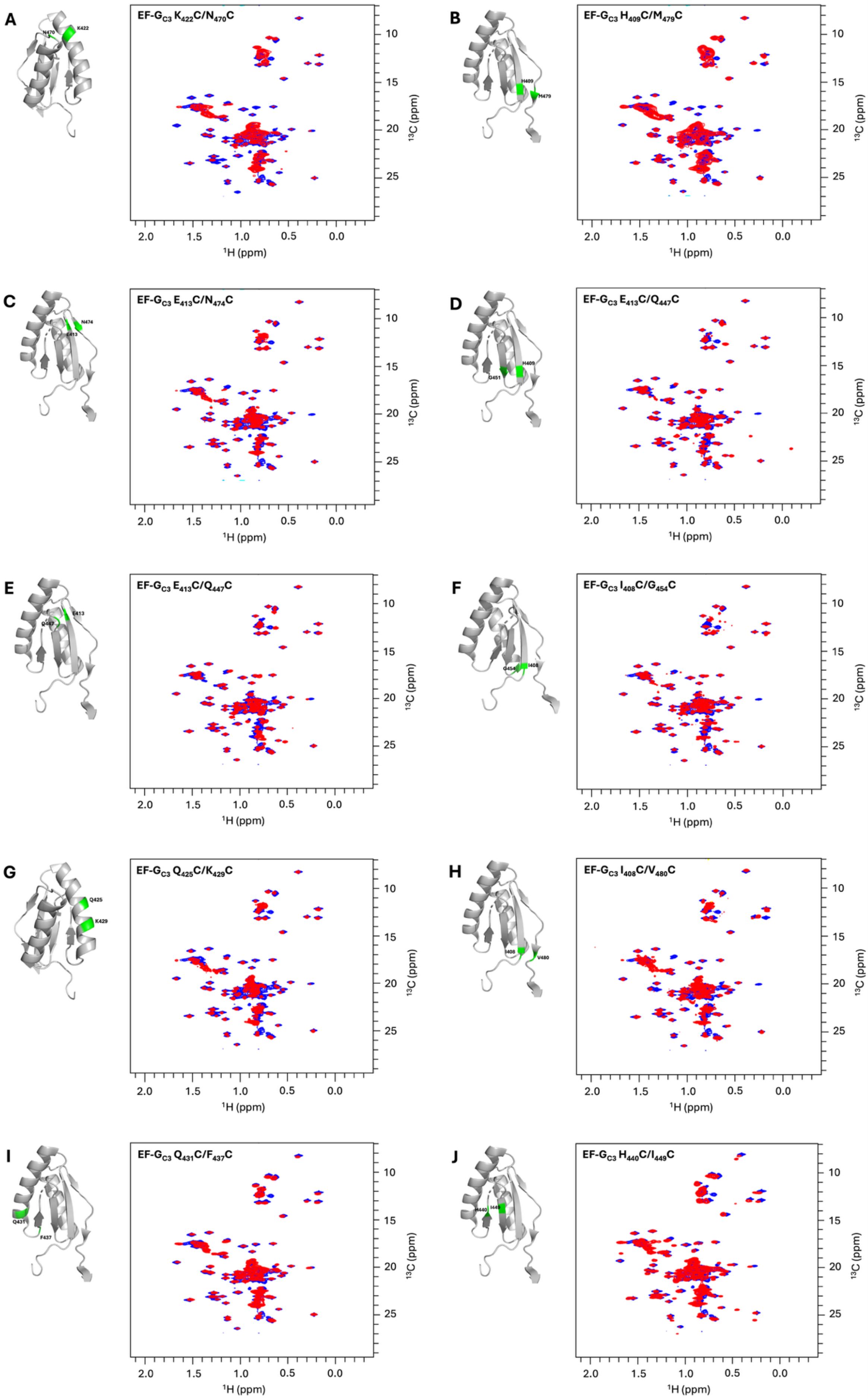

A
EF-G$_{C3}$ K$_{422}$C/N$_{470}$C
B
EF-G$_{C3}$ H$_{409}$C/M$_{479}$C
C
EF-G$_{C3}$ E$_{413}$C/N$_{474}$C
D
EF-G$_{C3}$ E$_{413}$C/Q$_{447}$C
E
EF-G$_{C3}$ E$_{413}$C/Q$_{447}$C
F
EF-G$_{C3}$ I$_{408}$C/G$_{454}$C
G
EF-G$_{C3}$ Q$_{425}$C/K$_{429}$C
H
EF-G$_{C3}$ I$_{408}$C/V$_{480}$C
I
EF-G$_{C3}$ Q$_{431}$C/F$_{437}$C
J
EF-G$_{C3}$ H$_{440}$C/I$_{449}$C
^{1}H (ppm)
^{13}C (ppm)

Figure S3. ^{1}H-^{13}C-HMQC spectra for variants of EF-G_{C3} bound to FusB (red) are shown overlaid with spectra of WT EF-G_{C3}:FusB (blue), showing all spectra overlay well with the WT spectrum, indicating that FusB binds to all variants and there are no significant structural differences. All spectra are displayed with the locations of the amino acid substitutions labelled on the structure of EF-G domain III. **(A)** Variant $K_{422}C/N_{470}C$. **(B)** Variant $H_{409}C/M_{479}C$. While all bound state peaks are observed and no apo EF-G_{C3} peaks are seen for these two variants, there is a slight suggestion of some additional disorder, which may be due to a higher population of the more disordered minor state for these variants as seen for ^{1}H-^{15}N spectra and discussed in the text. **(C)** Variant $E_{413}C/N_{474}C$. **(D)** Variant $H_{409}C/G_{451}C$. Some domain III peaks that broaden upon FusB binding in the WT are visible in this variant. **(E)** Variant $E_{413}C/Q_{447}C$. Some peaks in domain III are visible in the apo position, as discussed in the text but no apo peaks are visible in the binding site so this is not reflective of incomplete binding of FusB. **(F)** Variant $I_{408}C/G_{454}C$. The only differences observed in the variant spectrum are as discussed in the text and reflect changes due to changes in FA resistance. **(G)** Variant $Q_{425}C/K_{429}C$. **(H)** Variant $I_{408}C/V_{480}C$. **(I)** Variant $Q_{431}C/F_{437}C$. **(J)** Variant $H_{440}C/I_{449}C$.

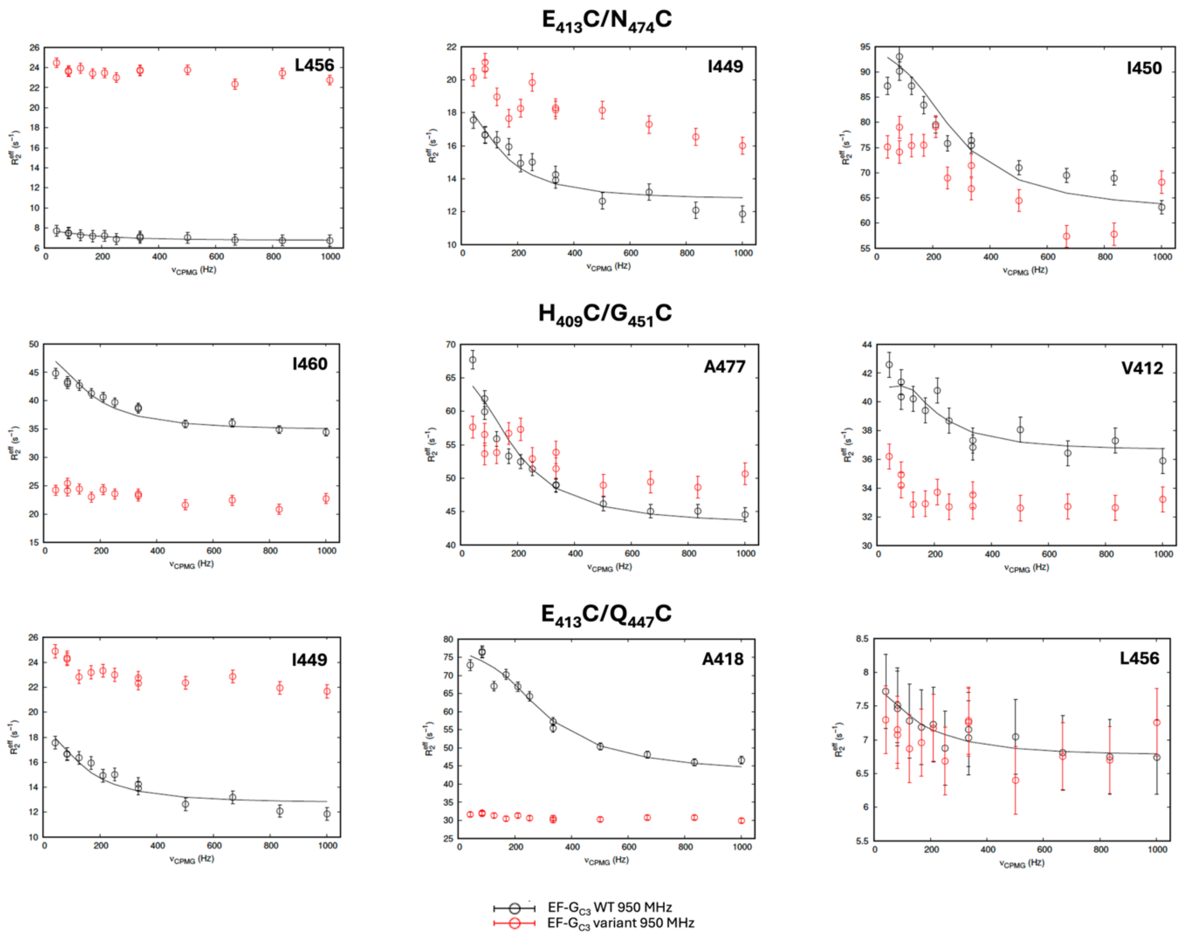

Figure S4. Comparison of relaxation dispersion profiles at 950 MHz within domain III between WT EF-G_{C3} (black) and variants $H_{409}C/G_{451}C$, $E_{413}C/Q_{447}C$ and $E_{413}C/N_{474}C$ (red) when bound to FusB.

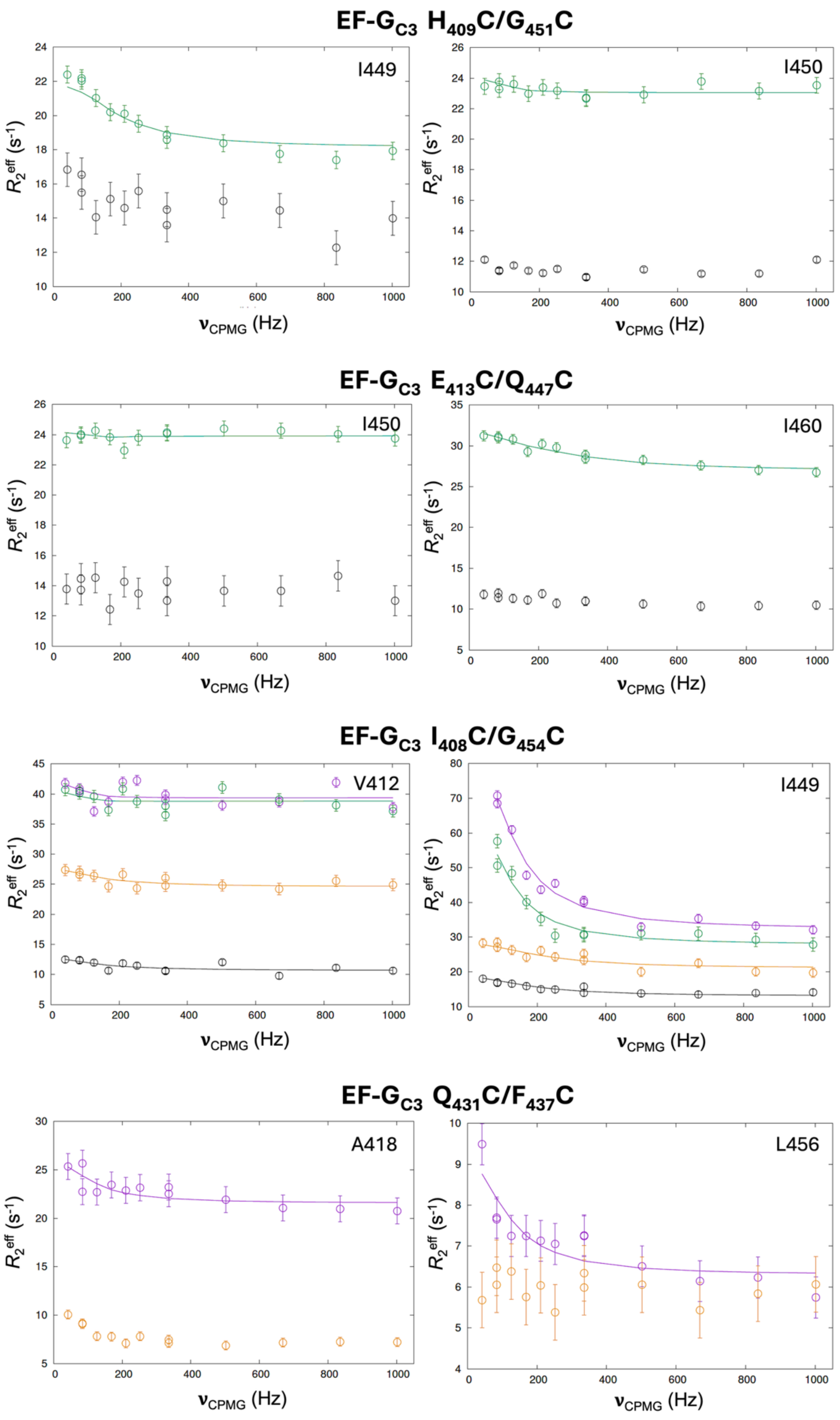

Figure S5. Comparison of relaxation dispersion profiles within domain III between EF-G_{C3} variants in the absence of FusB at 950MHz (orange) or 750 MHz (black) with the same variants bound to FusB at 950 MHz (purple) or 750 MHz (green). Variants $H_{409}C/G_{451}C$ and $E_{413}C/Q_{447}C$ restrain the central β-strands and show little relaxation

dispersion in either state, with some small increases in dispersion when bound to FusB, consistent with FusB increasing the minor state relative to the apo protein. Variant $I_{408}C/G_{454}C$ restrains the second α-helix and shows some dispersion effects in the apo state, which increase when bound to FusB, reflecting a higher population of the minor state in the apo protein compared with WT which is increased further in response to FusB binding. Variant $Q_{431}C/F_{437}C$ shows a slight increase in dispersion effects overall upon FusB binding, consistent with a smaller increase in minor state population for this variant, although residue I_{449} shows an increased dispersion profile in the apo state.

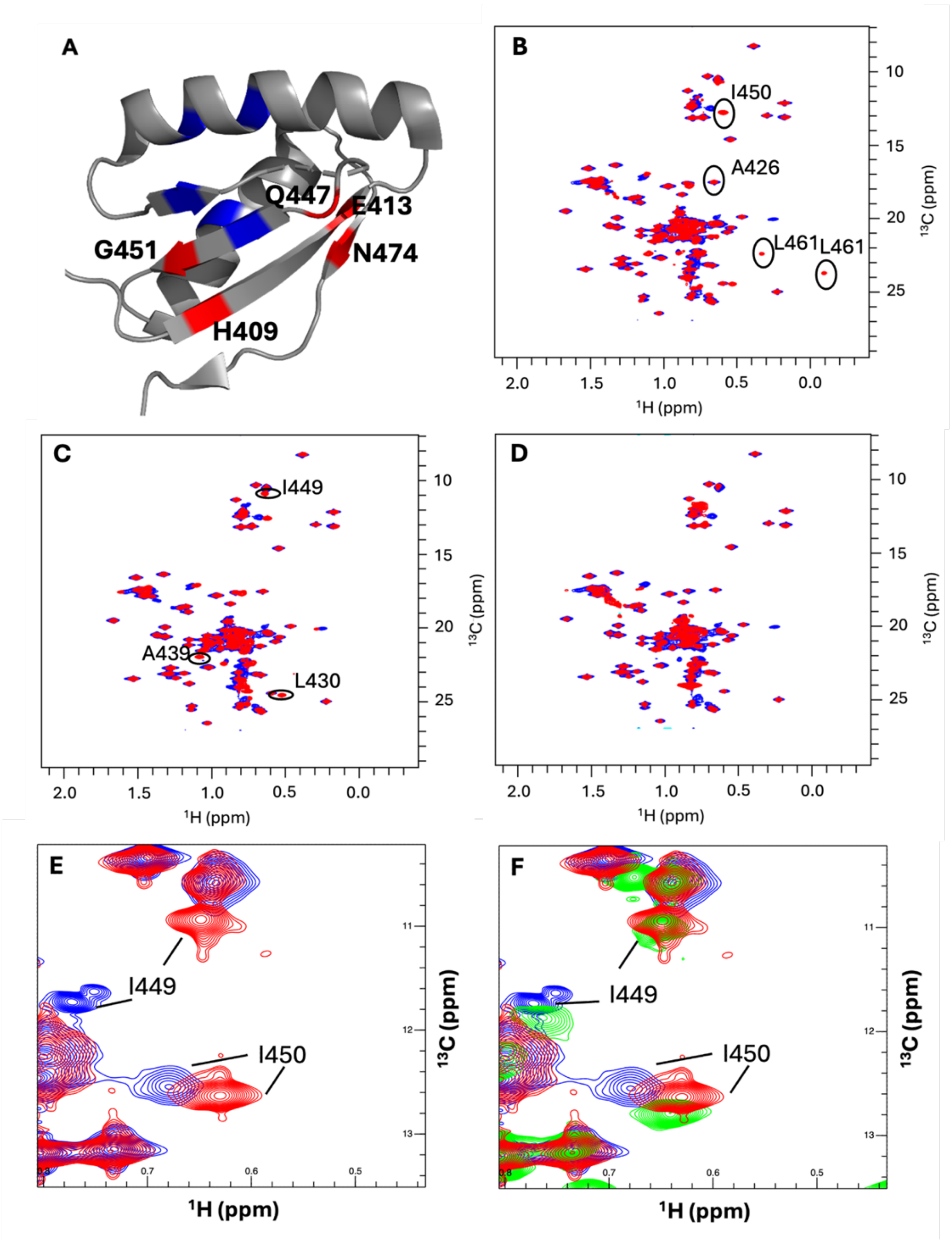

Figure S6. (A) The locations in EF-G domain III of amino acid substitutions for variants $H_{409}C/G_{451}C$, $E_{413}C/Q_{447}C$ and $E_{413}C/N_{474}C$ (red) and residues A_{426}, L_{430}, A_{439}, I_{449}, I_{450} and L_{461} (blue) which show chemical shift changes or loss of broadening in ^{1}H-^{13}C-HMQC methyl spectra for variants compared with WT EF-G_{C3}. **(B, C and D)** ^{1}H-^{13}C-HMQC spectra for variants $H_{409}C/G_{451}C$ **(B)**, $E_{413}C/Q_{447}C$ **(C)** and $E_{413}C/N_{474}C$ **(D)** bound to FusB (red) are shown overlaid with spectra of WT EF-G_{C3}:FusB (blue), showing overall few chemical shift changes. This shows none of the substitutions prevent FusB binding. Notable changes are the appearance of apo resonances of residues A_{426}, L_{430}, A_{439}, I_{449}, I_{450} and L_{461} and loss of broadening of I_{450} as well as shifting of I_{449} and I_{450} resonances in $H_{409}C/G_{451}C$ and $E_{413}C/Q_{447}C$

variants. **(E and F)** Zoomed view of the I_{449} and I_{450} region showing overlays of the ^{1}H-^{13}C spectra of WT EF-G_{C3} bound to FusB (blue) and the EF-G_{C3} $E_{413}C/Q_{447}C$ variant bound to FusB (red) as well as **(F)** the WT EF-G_{C3} apo spectrum (green) showing movement of the I_{449} and I_{450} peaks for the $E_{413}C/Q_{447}C$ variant towards the apo position.

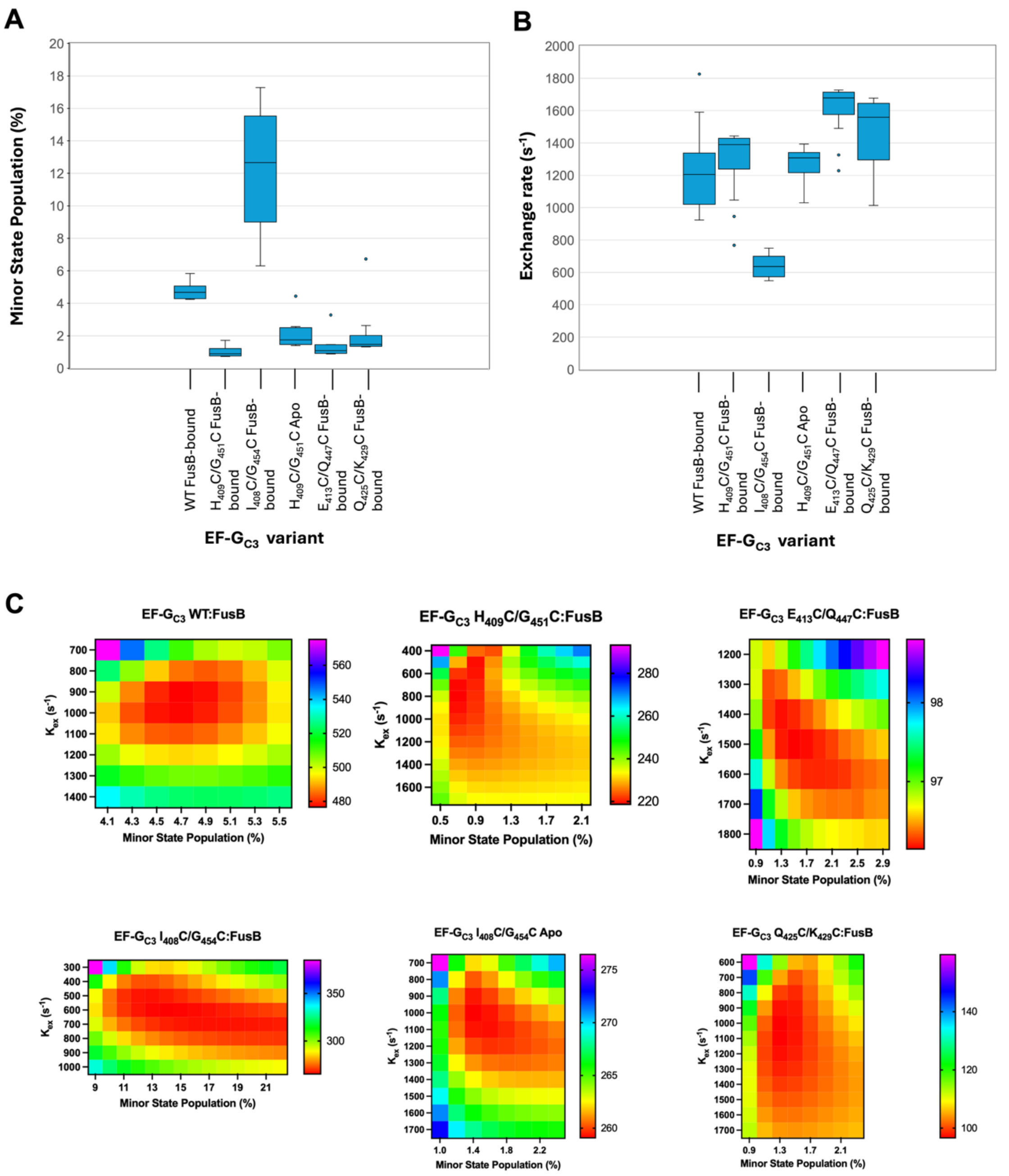

Figure S7. Plots of the range of fitted minor state populations (**A**) and exchange rates (**B**) for EF-G$_{C3}$ variants for which relaxation dispersion data was acquired at 2 field strengths. Fitting was repeated for the same data fixing either the exchange rate (**A**) or minor state population (**B**) at a range of values and the corresponding fitted parameter was recorded. Plots show the variation in the fitted minor state population over the range of exchange rates 100-1900 s^{-1} or the variation in the exchange rate over the range of minor state populations 0.2-8 %, except for EF-G$_{C3}$ I$_{408}$C/G$_{454}$C in the presence of FusB for which the minor state was varied over 2-20 %. Plots show that the fitted minor state population is not dependent on the

exchange rate and the fitted exchange rate is not dependent on the minor state population. (**C**) χ^2 values for fitting relaxation dispersion data determined at a range of fixed exchange rates and populations, showing minima focused on discrete points correlating with the fitted values, showing that the fitted parameters are well defined. The bar to the right of each plot indicates the scale for χ^2 values for that combination of K_{ex} and minor state population in each case.

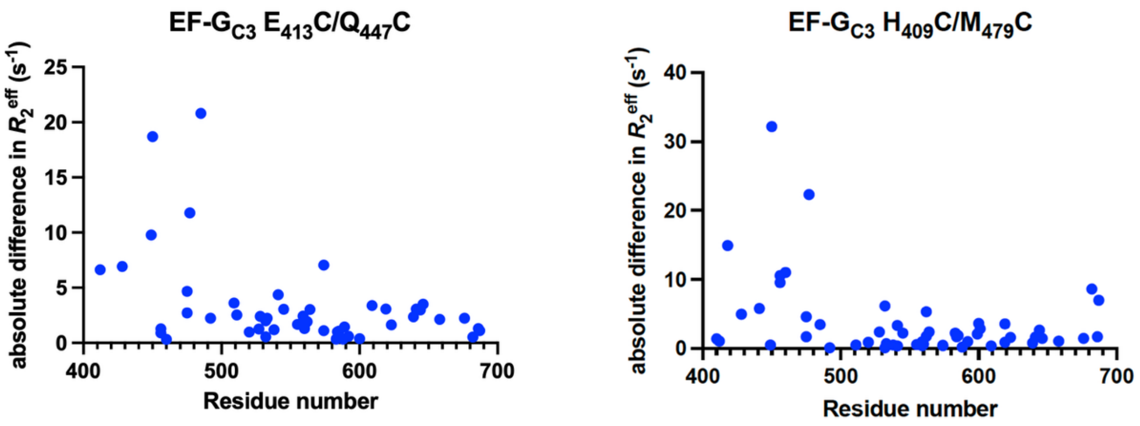

Figure S8. Absolute difference in R_2^{eff} plateau values between mutant and wild type proteins bound to FusB for EF-G$_{C3}$ E$_{413}$C/Q$_{447}$C, which shows a loss of FusB mediated fusidic acid resistance and a loss of FusB-mediated increase in domain III minor state, and EF-G$_{C3}$ H$_{409}$C/M$_{479}$C, which shows an increase in the minor state of domain III in the presence and absence of FusB. Values were determined by calculating the average R_2^{eff} for the last 3 points of each curve and calculating the absolute difference between these values for wild type and mutant proteins. The large variation is concentrated in domain III (residues 401-482), where there is a change in the conformational flexibility, rather than domains IV and V, containing the FusB binding site.

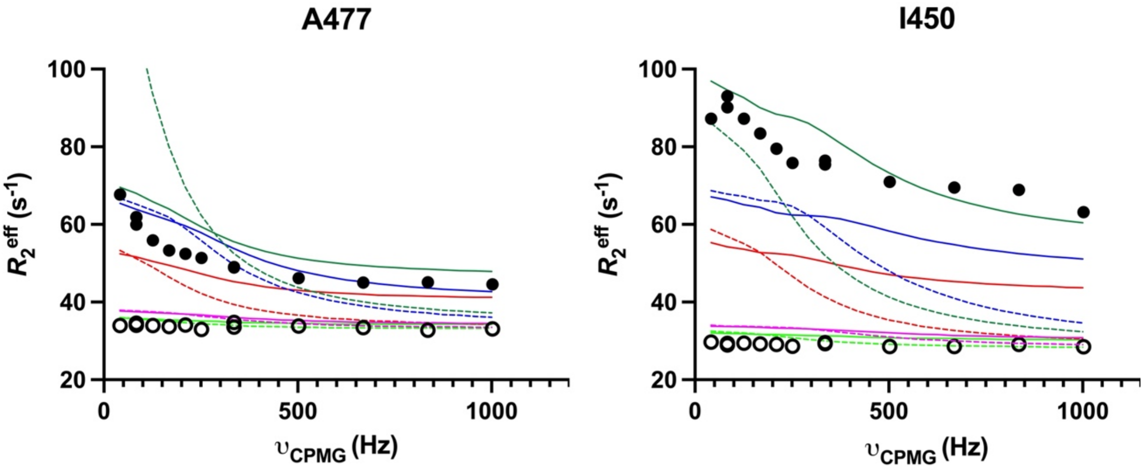

Figure S9. Simulations of multi-quantum CPMG relaxation dispersion data showing differences in R_2^{eff} plateau values (R_2^{inf}) caused by different values for the difference in chemical shifts and minor state population. Relaxation dispersion data at 950 MHz for WT EF-G_{C3} (filled circles) and EF-G_{C3} $E_{413}C/Q_{447}C$ (open circles) in the presence of FusB are compared to simulated curves using the R_2 apparent from the EF-G_{C3} $E_{413}C/Q_{447}C$ R_2^{inf}, the R_{ex} determined previously for WT EF-G_{C3} bound to FusB[16] and varied chemical shift differences and minor state populations. When the ^1H chemical shift difference is set to 0.02 ppm (dotted lines), the R_2^{eff} tends to the plateau value seen in EF-G_{C3} $E_{413}C/Q_{447}C$ regardless of the ^{13}C chemical shift difference or minor state population. When the ^1H chemical shift difference is increased (solid lines) to either 0.08 ppm (A477) or 0.12 ppm (I450), the curves remain similar for a population of 0.7 % (light green and magenta) but the R_2^{inf} is raised for higher minor state populations. Increasing the ^{13}C chemical shift difference from that observed in WT EF-G_{C3} data[16] (light green, pb = 0.7 %, red, pb = 4.7 % and dark green, pb = 8.7 %) to 1.15 ppm (A477) or 1.8 ppm (I450) (magenta, pb = 0.7% and blue, pb = 4.7 %) the magnitude of the dispersion curve is increased for the higher minor state population but shows no significant effect for a minor state population of 0.7 %. Differences in ^1H chemical shift difference alongside differences in the minor state population can therefore account for the differences observed in R_2^{inf} values. Error bars for experimental data are smaller than the markers and therefore not visible.

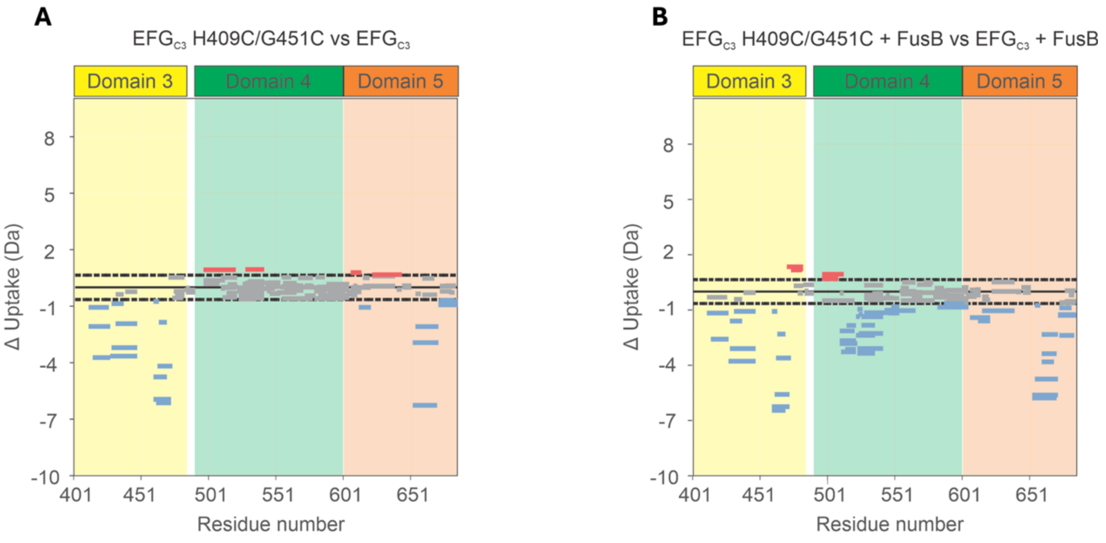

Figure S10. Wood's plots showing the summed differences in deuterium uptake when comparing WT EF-G_{C3} with EF-G_{C3} $H_{409}C/G_{451}C$ in **(A)** the absence and **(B)** the presence of FusB. This figure was generated using Deuteros[36]. Peptides coloured in blue or red, respectively, are protected or deprotected from exchange in the variant compared to the WT protein. Peptides with no significant difference between conditions, using a 98% confidence interval (dotted line), are shown in grey. In each case, domain III (yellow shading) shows protection throughout the domain, suggesting that the domain III minor state is less populated in the variant in both the presence and absence of FusB.

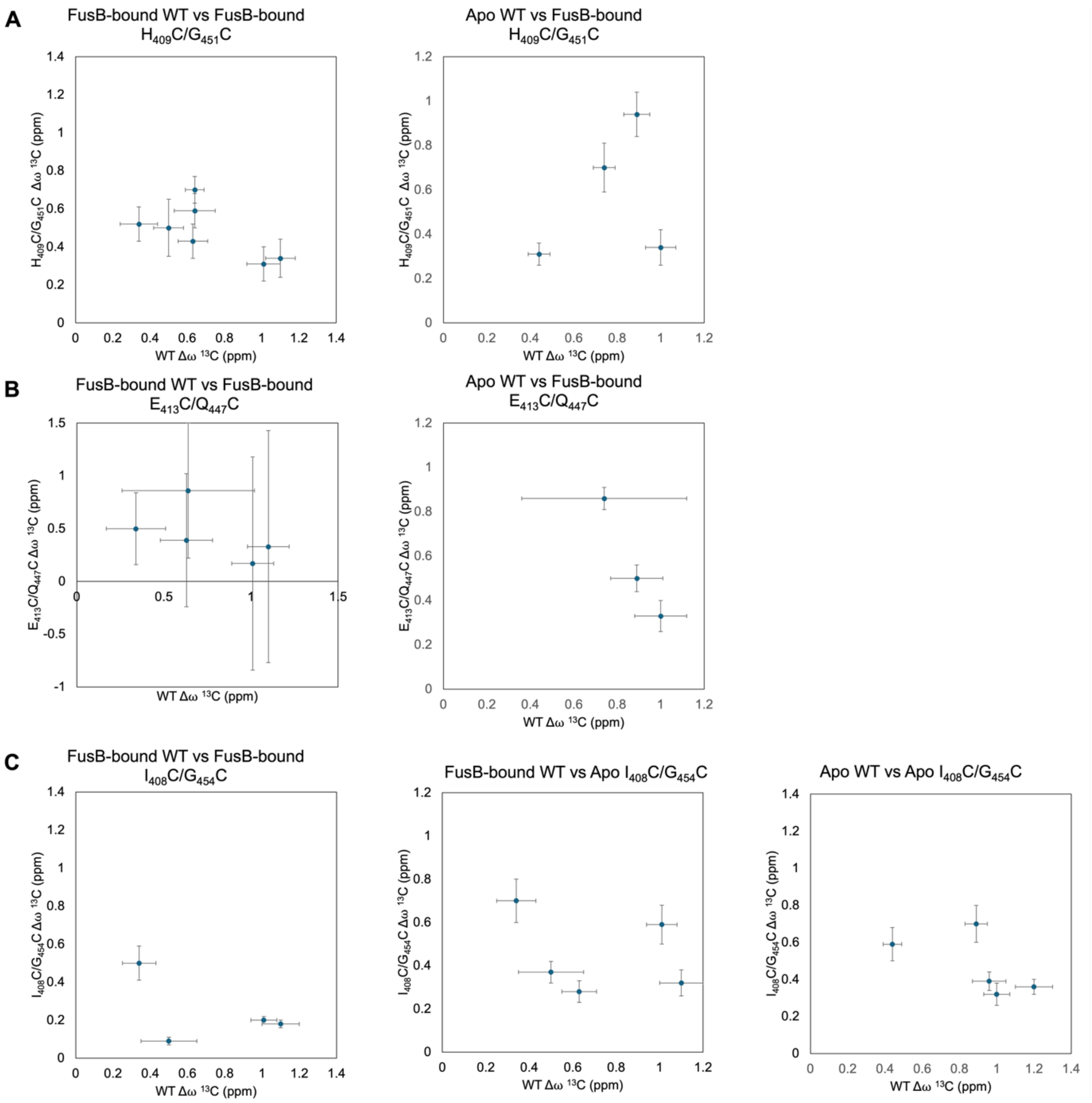

Figure S11. Comparisons of differences in ^{13}C chemical shifts between major and minor states determined from fitting relaxation dispersion data for WT and **(A)** EF-G_{C3} $H_{409}C/G_{451}C$, **(B)** EF-G_{C3} $E_{413}C/Q_{447}C$ and **(C)** EF-G_{C3} $I_{408}C/G_{454}C$. In **A** and **B** FusB-bound variant is compared to both FusB-bound and apo WT as the FusB-bound variant minor state has a similar population to the WT apo protein. In **C** the FusB-bound variant is compared to the FusB-bound WT and the apo variant is compared to the apo WT. However, the apo variant is also compared to the FusB-bound WT because the population of the variant in the absence of FusB is increased relative to the WT apo protein. The plots show poor correlations suggesting that the minor states, while similar to the WT in that there is increased disorder throughout domain III, may occupy different conformations for the different variants. However, the varying effects of ^{1}H chemical shift differences on accurate fitting of ^{13}C values at different minor state populations reduces the accuracy with which such conclusions can be drawn.

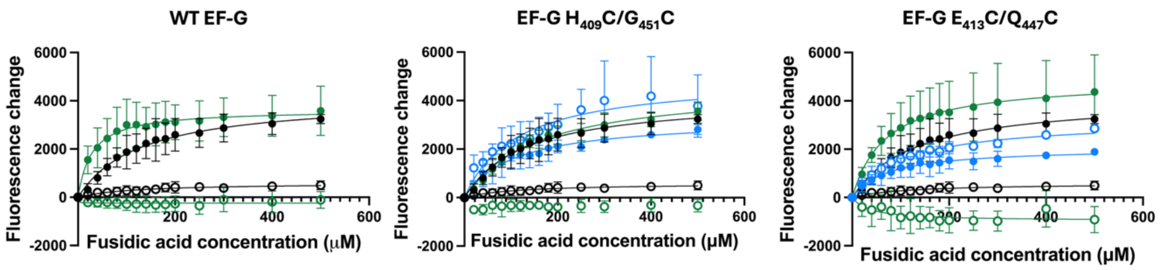

Figure S12. Comparison of FA stalling assays for EF-G $H_{409}C/G_{451}C$ and EF-G $E_{413}C/Q_{447}C$ in the presence (green) and absence (blue) of 5 mM DTT. In each case, the WT data in the absence of DTT are shown in black. Assays were repeated in the absence (closed circles) and presence (open circles) of 5 X molar excess of FusB. The addition of 5mM DTT makes no significant difference to the fluorescence observed for the WT protein (left panel) but results in an increase in fluorescence in the absence of FusB for both variants, indicating the disulphide bonds hindered correct ribosome binding. The build-up of fluorescence seen in the presence of FusB for both variants is lost in the presence of DTT, showing that the loss of resistance was due to the disulphide bonds.

121

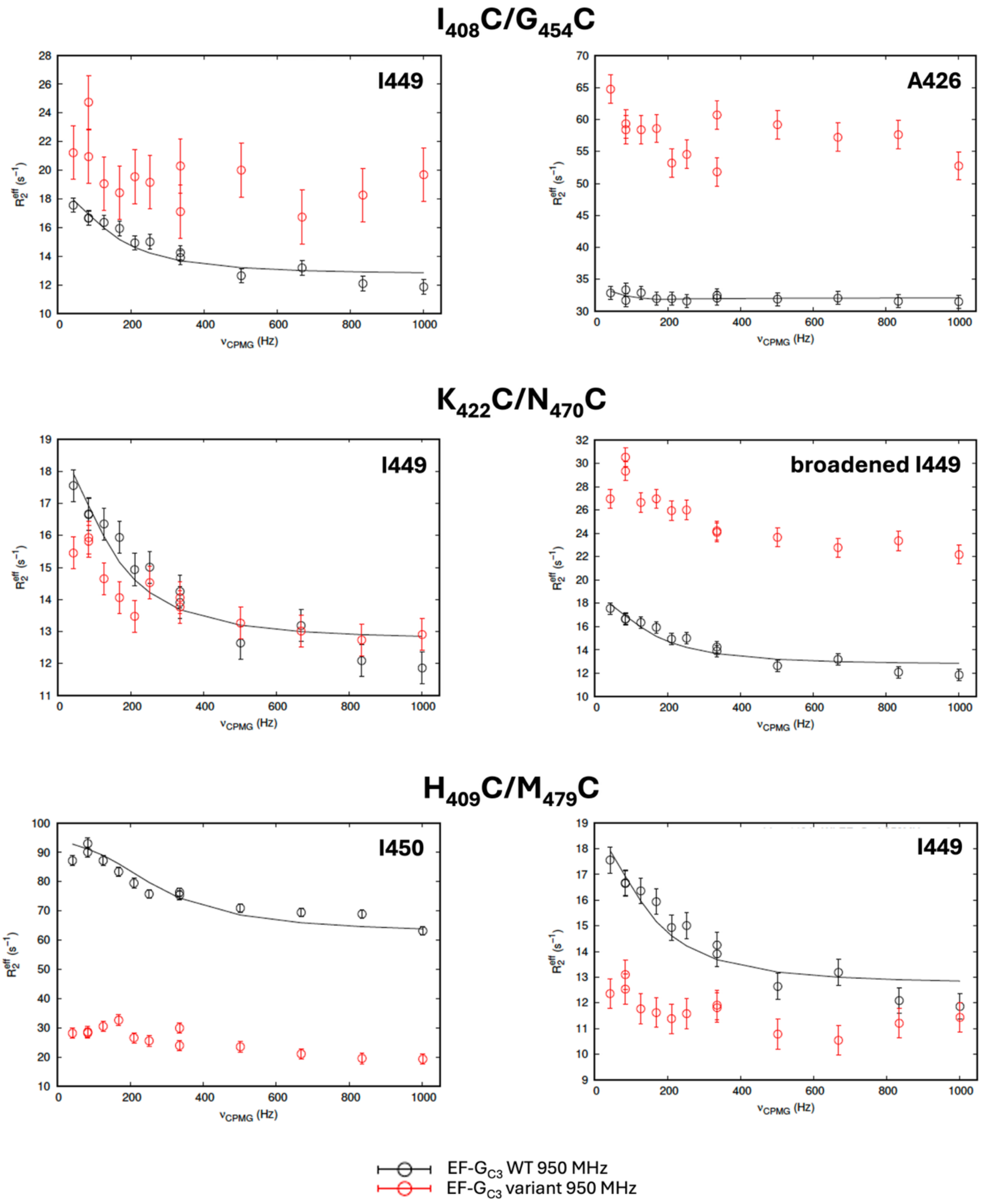

Figure S13. Comparison of relaxation dispersion profiles at 950 MHz within domain III between WT EF-G (black) and variants $I_{408}C/G_{454}C$, $K_{422}C/N_{470}C$ and $H_{409}C/M_{479}C$ (red) when bound to FusB. Although most residues show a decrease in dispersion when compared to WT, some residues, such as A_{426} in variant $I_{408}C/G_{454}C$ show an increase in dispersion.

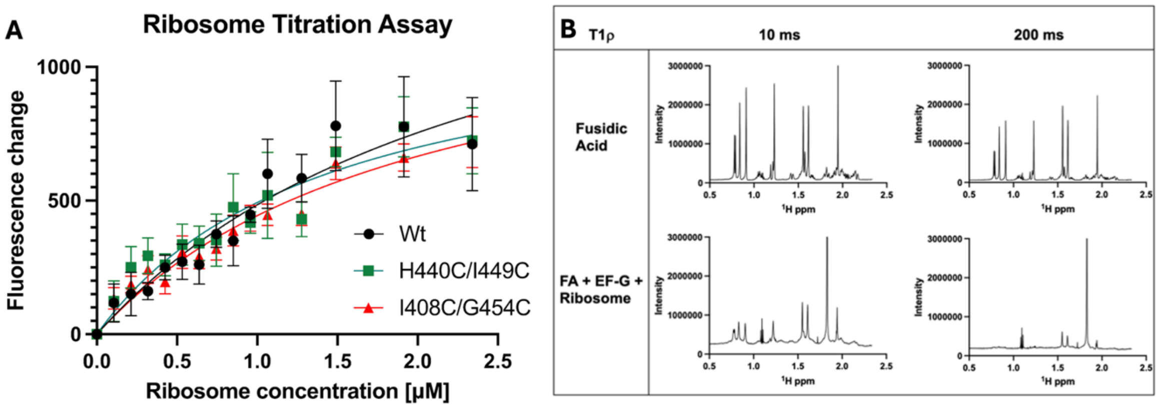

Figure S14. (A) Formation of ribosome:EF-G:GDP complexes upon titration of ribosomes. Variants $H_{440}C/I_{449}C$ and $I_{408}C/G_{454}C$ bind to ribosomes comparably to WT EF-G. **(B)** *T1ρ* experiments with spin-locking for 10 ms and 200 ms of FA in the presence and absence of EF-G $I_{408}C/G_{454}C$:ribosome complexes. FA alone serves as a reference for the unbound state. A decrease in intensity for FA + EF-G $I_{408}C/G_{454}C$ + ribosome is observed when compared to FA alone and confirms FA binding.

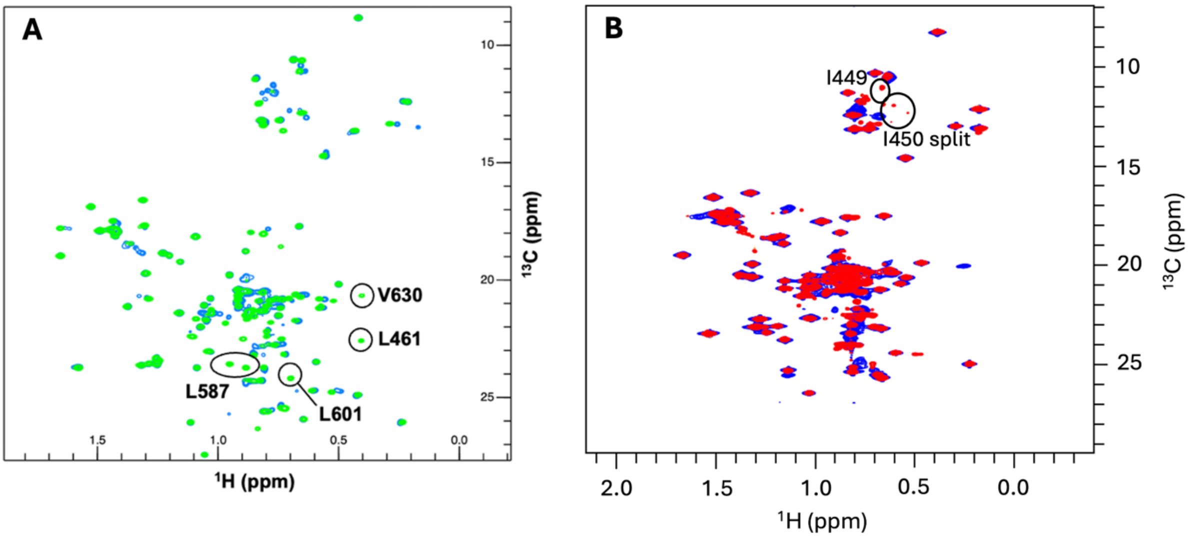

Figure S15. (A) ^{1}H-^{13}C-HMQC spectrum of variant EF-G_{C3} $I_{408}C/G_{454}C$ in the apo state (light blue) overlaid with apo WT EF-G_{C3} (green). **(B)** ^{1}H-^{13}C-HMQC spectra for variant EF-G_{C3} $I_{408}C/G_{454}C$ bound to FusB (red) are shown overlaid with spectra of WT EF-G_{C3}:FusB (blue). Notable changes are the appearance of apo resonances of residues $A_{426,}$ L_{430}, A_{439} and I_{449}, as well as the splitting of I_{450} and I_{460} in FusB bound spectra, and the disappearance of resonances from the apo spectrum of variant $I_{408}C/G_{454}C$ of residues L_{461}, L_{587}, V_{601} and V_{630}. Residue V_{601} is located in the region connecting domains III and IV and IV and V found to be important for relative rearrangements of domains during binding and unbinding of the ribosome[4] and residues L_{587} and V_{630} are oriented towards the ribosome binding interface.

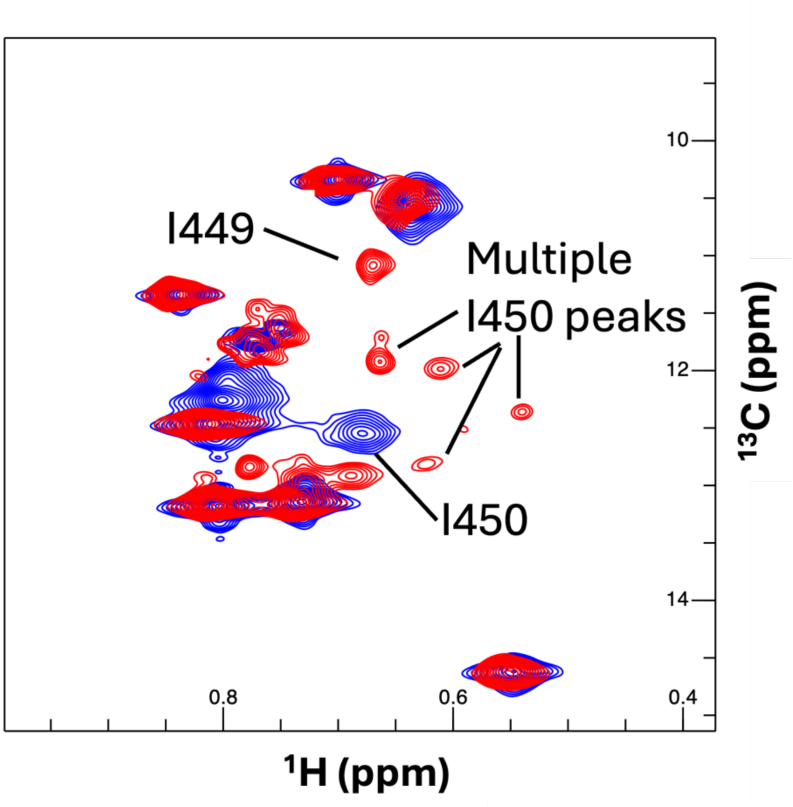

Figure S16. The region surrounding I_{450} in the ^{1}H-^{13}C HMQC spectrum of WT EF-G_{C3} (blue) and EF-G_{C3} $I_{408}C/G_{454}C$ (red) bound to FusB, showing the I_{450} peak is replaced by multiple peaks in the mutant spectrum.

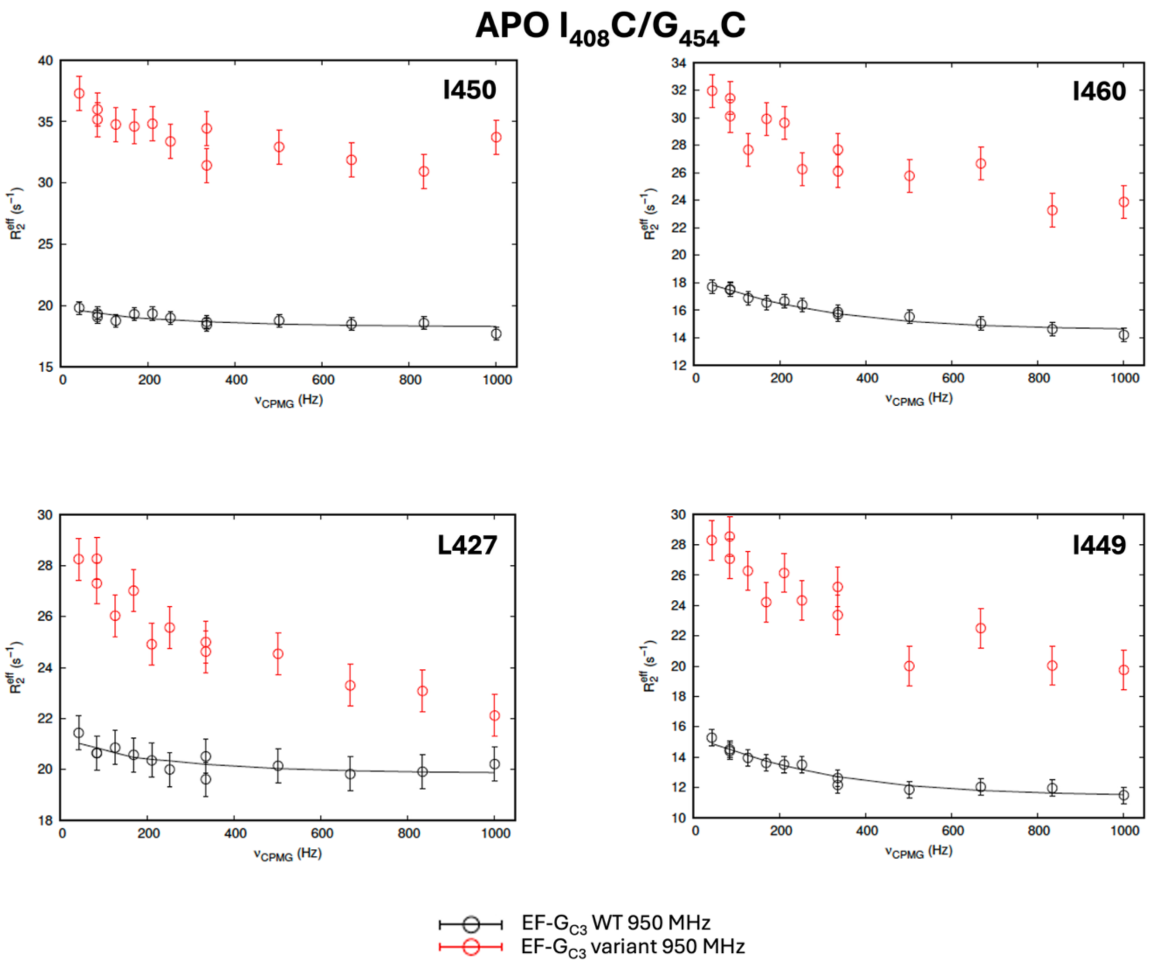

Figure S17. Comparison of relaxation dispersion profiles at 950 MHz of apo EF-G_{C3} variant $I_{408}C/G_{454}C$ (red) and apo WT EF-G_{C3} (black) showing increased dispersion profiles in the variant compared to the WT protein.

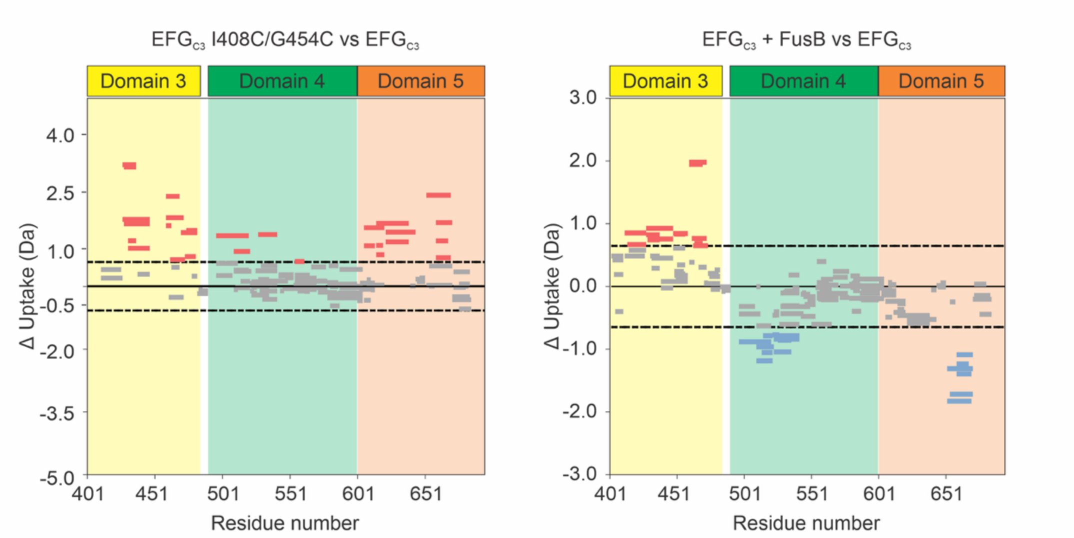

Figure S18. Wood's plot showing the summed differences in deuterium uptake (see Methods) when comparing (left) WT EF-G$_{C3}$ vs EF-G$_{C3}$ I$_{408}$C/G$_{454}$C, or (right) WT EF-G$_{C3}$ upon FusB binding (reproduced from **Figure 3A** in the main text). In the variant protein, domain III shows increased deprotection relative to the WT protein, similar to that seen in WT EF-G$_{C3}$ upon FusB binding (right), showing that the minor state, which is increased in the variant, is similar to that observed upon FusB binding.

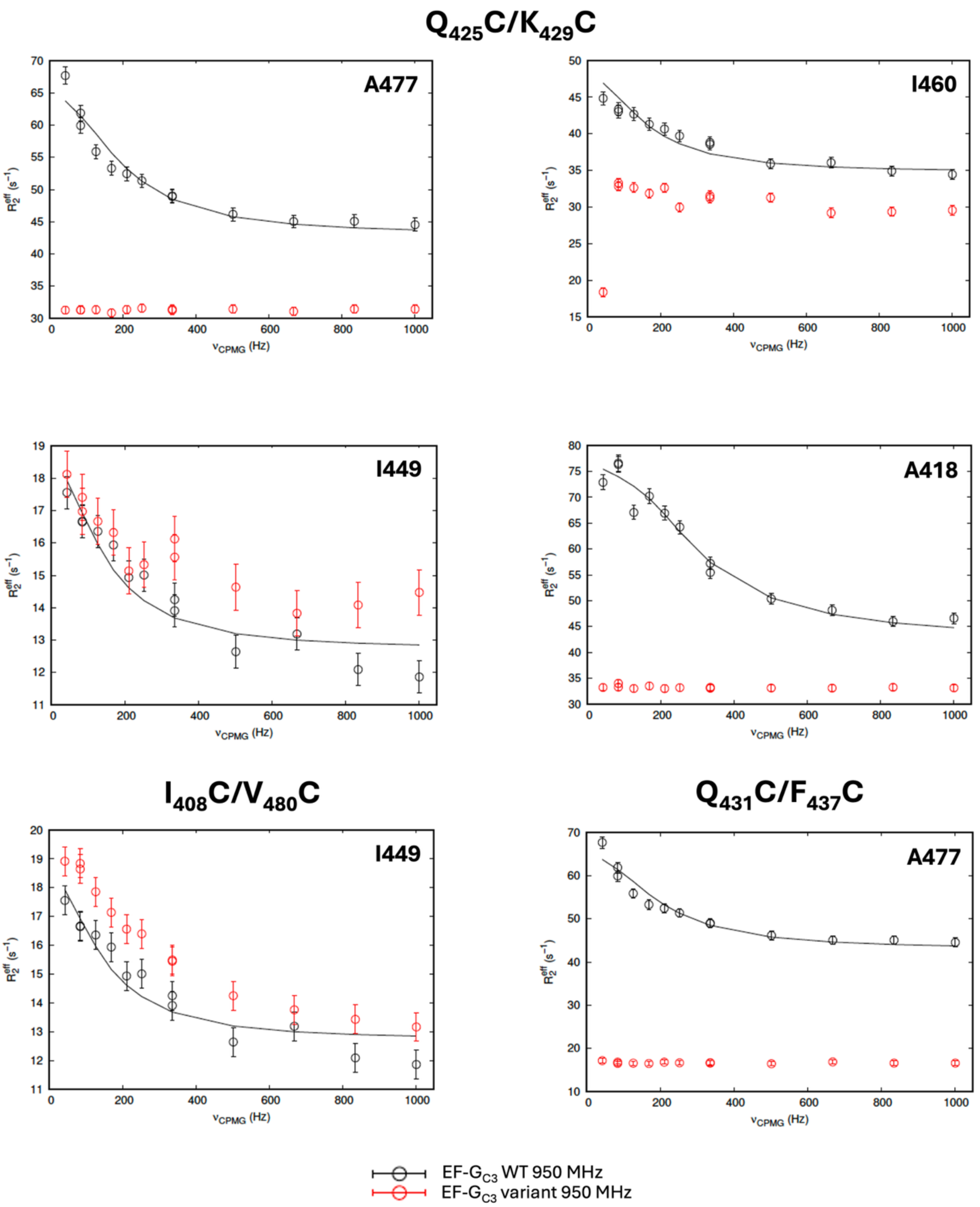

Figure S19. Comparison of relaxation dispersion profiles at 950 MHz of EF-G_{C3} variants $Q_{425}C/K_{429}C$, $I_{408}C/V_{480}C$ and $Q_{431}C/F_{437}C$ (red) and WT EF-G_{C3} (black) bound to FusB. Despite an overall reduction in dispersion for these variants when compared to WT, dispersion is preserved and approximately the same as in WT for residue I_{449} for variants $Q_{425}C/K_{429}C$ and $I_{408}C/V_{480}C$.

Table S1. Summary of major findings and groupings as depicted in figure 7.

**EF-G variant**	**Disulphide bond location**	**Change in FusB induced dynamics**	**Change in apo dynamics**	**Resistant to FA?**	**FusB confers resistance?**	**Group**
$E_{413}C/Q_{447}C$	Between β-strands	Dynamics reduced comparable to apo WT	N/A	No	No	A: β-sheet stablising Prevents FusB resistance
$H_{409}C/G_{451}C$	Between β-strands	Dynamics reduced comparable to apo WT	N/A	No	No	A: β-sheet stablising Prevents FusB resistance
$E_{413}C/N_{474}C$	Between β-strands	Dynamics reduced comparable to apo WT	N/A	No	No	A: β-sheet stablising Prevents FusB resistance
$I_{408}C/G_{454}C$	α-helix 2 to β-strand	Dynamics increased within β-sheet, reduced elsewhere	Dynamics increased within β-sheet	Yes	Yes	B: β-sheet destablising Confers resistance without FusB
$K_{422}C/N_{470}C$	α-helix 2 to α-helix 1	Dynamics increased within β-sheet, reduced elsewhere	N/A	Yes	Yes	B: β-sheet destablising Confers resistance without FusB
$H_{409}C/M_{479}C$	Linker to domain IV to β-strand	Dynamics increased within β-sheet, reduced elsewhere	N/A	Yes	Yes	B: β-sheet destablising Confers resistance without FusB

$Q_{425}C$/ $K_{429}C$	Within α-helix 1	Dynamics reduced but not abolished L_{456} shows increased dyanmics	N/A	No	Yes	C: No change in resistance modest minor state population increase only
$I_{408}C$/ $V_{480}C$	Between linker to domain II and linker to domain III	Dynamics reduced but not abolished L_{456} shows increased dyanmics	N/A	No	Yes	C: No change in resistance modest minor state population increase only
$Q_{431}C$/ $F_{437}C$	α-helix 1 to β-strand	Dynamics reduced but not abolished L_{456} shows increased dyanmics	N/A	No	Yes	C: No change in resistance modest minor state population increase only
$H_{440}C$/ $I_{449}C$	Between β-strand 2 and linker following β-strand 1	Dynamics greatly reduced but not abolished	N/A	Partially	Yes	D: ribosome stalling is reduced in the absence of FusB but FusB can confer further resistance

Table S2. The residues included in CPMG relaxation dispersion fitting for each variant. Although WT data were derived from an earlier study[2], the residues included in the fit for WT are also listed for comparison.

Fitted Variant	Residues Included in Fit
WT EF-G_{C3} apo	I_{408}, A_{418}, I_{449}, I_{450}, I_{460}, V_{462}, V_{471}
WT EF-G_{C3} FusB-bound	V_{412}, A_{418}, L_{427}, V_{448}, I_{449}, I_{450}, I_{460}, A_{477}
EF-G_{C3} $H_{409}C/G_{451}C$ FusB-bound	L_{410}, V_{412}, A_{418}, I_{449}, I_{450}, L_{456}, I_{460}, A_{477}
EF-G_{C3} $E_{413}C/Q_{447}C$ FusB bound	A_{418}, I_{449}, I_{450}, L_{456}, I_{460}, A_{477}
EF-G_{C3} $I_{408}C/G_{454}C$ apo	L_{410}, V_{412}, A_{418}, V_{428}, A_{439}, I_{449}, I_{450}, L_{456}, V_{462}, A_{477}
EF-G_{C3} $I_{408}C/G_{454}C$ FusB-bound	L_{410}, V_{412}, A_{418}, I_{449}, I449apo-like, I_{450}
EF-G_{C3} $K_{425}C/Q_{429}C$ FusB-bound	L_{410}, V_{428}, I_{449}, $I_{449apo\text{-}like}$, L_{456}

Table S3. The sequences of primers used in this study. Mis-matched nucleotides to introduce mutations are shown in bold.

Primer	Sequence	
FusA-H$_{409}$C/G$_{451}$C	H$_{409}$C	5'-TCAATGGAATTCCCAGAGCCAGTTATT**TGC**TTATCAGTAGAGCCA-3'
	G$_{451}$C	5'-CTGGACAAGTTATCATC**TGT**GGTATGGGTGAGCTT-3'
FusA-E$_{413}$C/Q$_{447}$C	H$_{413}$C	5'-CCAGAGCCAGTTATTCACTTATCAGTA**TGC**CCAAAATCTAAAGCTGACCAA-3'
	G$_{447}$C	5'-CACACTGACGAAGAAACTGGA**TGC**GTTATCATCGGTGGTATGGGT-3'
FusA-I$_{408}$C/G$_{454}$C	H$_{408}$C	5'-TGGAATTCCCAGAGCCAGTT**TGT**CACTTATCAGTAGAGCC-3'
	G$_{454}$C	5'-GTTATCATCGGTGGTA**TGT**GTGAGCTTCACTTAGACA-3'
FusA-Q$_{425}$C/K$_{429}$C	H$_{425}$C/ G$_{429}$C	5'-ATGGAATGTTGGGTCTTCTTCTTGTAA**GCA**AACTAAAGC**GCA**AGTCATTTTATCTTG-3'
FusA-K$_{422}$C/N$_{470}$C	K$_{422}$C	5'-TTATCAGTAGAGCCAAAATCTAAAGCTGACCCAAGATTGCATGACTCAAGCTTTAGTTA-3'
	N$_{470}$C	5'-CACCGTATGAAGAAAGAATTCTGCGTTGAATGTAACGTAGGTGC-3'
FusA-E$_{413}$C/N$_{474}$C	E$_{413}$C	5'-CCAGAGCCAGTTATTCACTTATCAGTATGCCCAAAATCTAAAGCTGACCAA -3'
	N$_{474}$C	5'-CCGTATGAAGAAAGAATTCAACGTTGAATGTGTTGCGTAGGTGCTCCATG -3'
FusA-H$_{409}$C/M$_{479}$C	H$_{409}$C	5'-TCAATGGAATTCCCAGAGCCAGTTATTTGCTTATCAGTAGAGCCA-3'
	M$_{479}$C	5'-CGTTGAATGTAACGTAGGTGCTCCATGGCGTTTCATATCGTGAAACATTCAAAT-3'
FusA-I$_{408}$C/V$_{480}$C	H$_{408}$C	5'-TGGAATTCCCAGAGCCAGTT**TGT**CACTTATCAGTAGAGCC-3'
	M$_{480}$C	5'-GAATGTAACGTAGGTGCTCCAA**TGT**GTTCATATCGTGAAACATTCAAATC-3'
FusA-Q$_{431}$C/F$_{437}$C	F$_{431}$C/ Q$_{437}$C	5'-GG**CAT**GTTGGGTCTTCTTCG**CAT**AATTTAACTAAAGCTTGAGTCATTTTATCTTGGT-3'
FusA-H$_{440}$C/I$_{449}$C	H$_{440}$C	5'-TCCAGTTTCTTCGTCAGTG**CAT**GCATGGAATGTTGGGTCT-3'
	I$_{449}$C	5'-CTCACCCATACCACCGAT**GCA**AACTTGTCCAGTTTCTTCG-3'
FusA-S2		5'-TGTGGTACAGCTTTCAAAAACAA-3'

SI References

1 Tomlinson, J. H., Thompson, G. S., Kalverda, A. P., Zhuravleva, A. & O'Neill, A. J. A target-protection mechanism of antibiotic resistance at atomic resolution: insights into FusB-type fusidic acid resistance. *Scientific Reports* **6**, 19524, doi:10.1038/srep19524 (2016).

2 Tomlinson, J. H., Kalverda, A. P. & Calabrese, A. N. Fusidic acid resistance through changes in the dynamics of the drug target. *Proceedings of the National Academy of Sciences* **117**, 25523-25531, doi:10.1073/pnas.2008577117 (2020).

3 Cox, G. *et al.* Ribosome clearance by FusB-type proteins mediates resistance to the antibiotic fusidic acid. *Proceedings of the National Academy of Sciences* **109**, 2102-2107, doi:10.1073/pnas.1117275109 (2012).

4 Li, W., Trabuco, L. G., Schulten, K. & Frank, J. Molecular dynamics of EF-G during translocation. *Proteins: Structure, Function, and Bioinformatics* **79**, 1478-1486, doi:https://doi.org/10.1002/prot.22976 (2011).

5 Lau AMC, A. Z. M. C. P. A. Deuteros: software for rapid analysis and visualization of data from differential hydrogen deuterium exchange-mass spectrometry. Bioinformatics 1, 3171–3173 (2019).
